# Mushrooms as potent autophagy modulators in cancer therapy: Current evidence and therapeutic prospects

**DOI:** 10.1016/j.cpt.2025.08.001

**Published:** 2025-08-21

**Authors:** Md. Mahmudul Hasan, Eva Azme, Rashedul Alam, Md. Jahirul Islam Mamun, Md. Tanvir Chowdhury, Md. Hossain Rasel, Md. Safayat Hossen Momen, Neamul Hoque, Md. Ekramul Haque Ekram, Nazmul Hasan Eshaque, Shakil Ahmed, Md. Tashrif Rahman Tipu, Sanjida Shahid Juthi, Mohammad Fazlul Kabir, Ahsan Ullah, Md. Liakot Ali, S.M. Moazzem Hossen, Hea-Jong Chung

**Affiliations:** a Department of Pharmacy, Faculty of the Biological Sciences, University of Chittagong, Chittagong 4331, Bangladesh; b Department of Biochemistry and Molecular Biology, Medical University of South Carolina, Charleston, SC 29425, USA; c Department of Biotechnology, Harrisburg University of Science and Technology, Harrisburg, PA 17101, USA; d Anderson Center for Autism, 4885 US-9, Staatsburg, NY 12580, USA; e Department of Chemistry and Biochemistry, Kennesaw State University, Georgia, GA 30144, USA; f School of Pharmacy, Institute of New Drug Development, Jeonbuk National University, Jeonju 54896, Republic of Korea; g Honam Regional Center, Korea Basic Science Institute (KBSI), Gwangju 61751, Republic of Korea; h College of Pharmacy, Chung-Ang University, Seoul 06974, Republic of Korea; i Department of Bio-Analysis Science, University of Science and Technology, Daejeon 34113, Republic of Korea

**Keywords:** Mushroom, Autophagy, Cancer, Traditional medicine, Functional food, Endoplasmic reticulum stress

## Abstract

Mushrooms, recognized for their culinary and medicinal applications, are emerging as promising autophagy modulators in cancer therapy. Autophagy is cellular degradation triggered by organelle damage, protein aggregation, metabolic disturbances, or nutrient scarcity. It contributes to the suppression of early tumor development and the promotion of cancer cell survival at advanced stages. This review systematically assesses the current evidence on the anticancer potential of mushrooms and their bioactive compounds, focusing on the ability of these mushrooms and their bioactive compounds to modulate autophagy. The review lists over 18 mushroom species (e.g., *Ganoderma lucidum [G. lucidum], Cordyceps, Phellinus) and 28 bioactive compounds (such as Ganoderic acid DM, Cordycepin, Hispidin*) that affect autophagy, demonstrating efficacy against 15 cancer types, including colorectal, lung, breast, and liver cancers. Essential compounds modulate autophagy through phosphoinositide 3-kinase (PI3K)/protein kinase B (Akt)/Mammalian target of rapamycin (mTOR), AMP-activated protein kinase (AMPK), and Beclin-1 pathways, resulting in notable anticancer effects. *G. lucidum* extracts significantly reduced colorectal tumor growth by up to 60% *in vivo*. Additionally, Cordycepin induced autophagic cell death in lung cancer cells, with IC_50_ values as low as 25 μmol/L. The findings highlight the potential of mushrooms as low-toxicity adjuvants to conventional therapies, providing additional advantages such as immune modulation and antioxidant activity. Mushrooms and their bioactive components present promising avenues for cancer therapy through the modulation of autophagy. The context-dependent effects of autophagy, along with the limited clinical evidence, present considerable challenges. Future clinical trials must focus on developing standardized extracts and personalized approaches to effectively translate this preclinical potential into clinical practice.

## Highlights

• Mushroom-derived compounds (e.g., Ganoderic Acid DM, Cordycepin) modulate autophagy through pathways such as phosphoinositide 3-kinase (PI3K)/protein kinase B (Akt)/Mammalian target of rapamycin (mTOR), AMP-activated protein kinase (AMPK) in 15 types of cancer.
• Mushroom-derived compounds target autophagy’s dual cancer role (early suppression, late promotion) for context-specific therapy.
• *Ganoderma lucidum and Antrodia cinnamomea* modulate autophagy preclinically, enhance chemosensitivity in colorectal, lung and breast cancer.
• Mushroom-derived compounds synergize with standard therapies via autophagy modulation.
• Clinical trials and preparation of standardized extracts are needed to use the mushroom’s autophagy potential in cancer therapies.

## Graphical abstract

**Figure:**
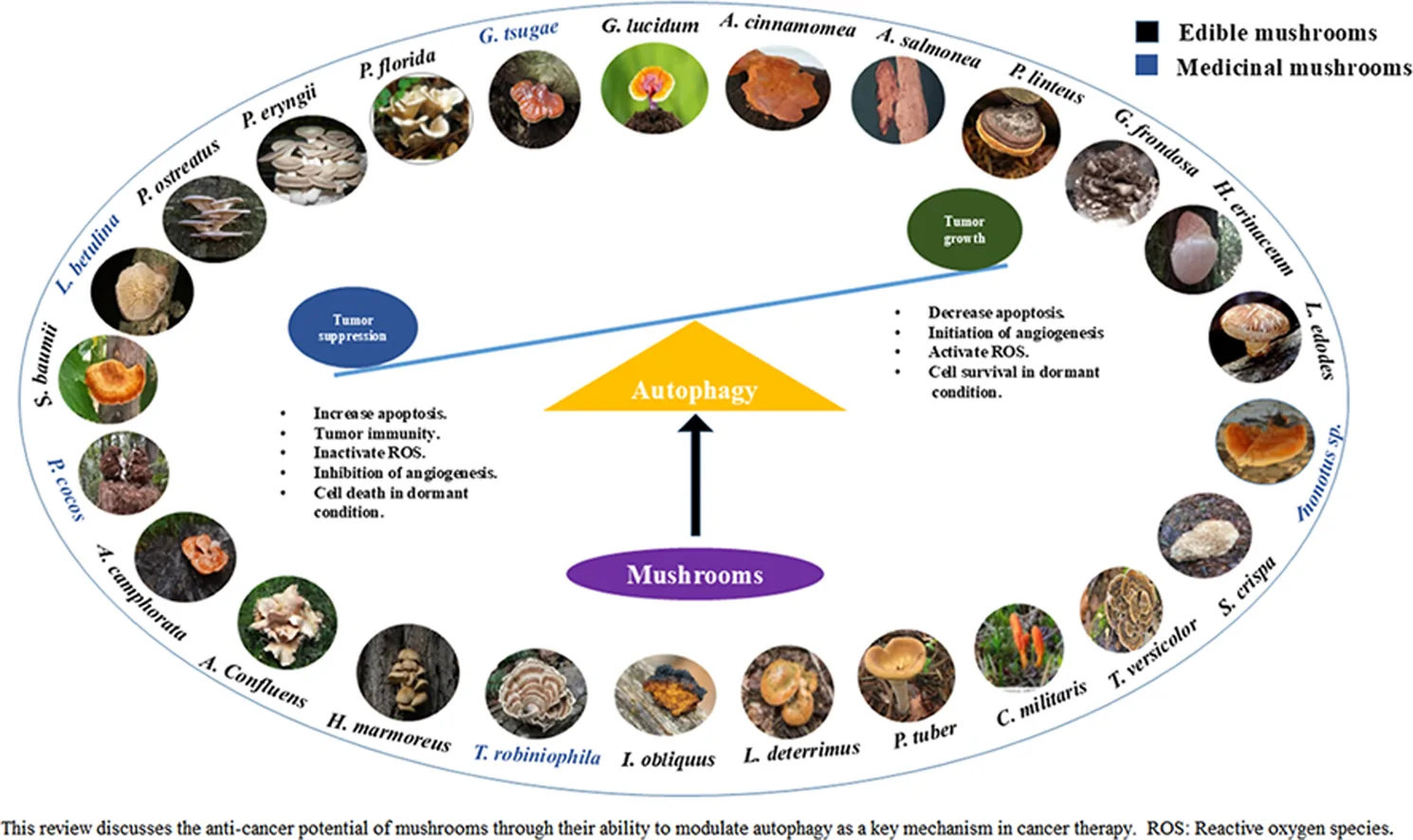

## Introduction

Mushrooms are valued for their culinary appeal and medicinal properties.^1^ Being rich in vitamins, minerals, and dietary fiber, they serve as an important component of a healthy diet.^2^ Moreover, mushrooms have been a core part of traditional Chinese medicine for centuries, due to their health-promoting benefits.^3^ *Ganoderma lucidum (G. lucidum)* (Reishi), *Lentinula edodes* (Shiitake), and *Grifola frondosa* (Maitake) are well-known for their immune-boosting, antioxidant, and anti-inflammatory properties.^4^, ^5^, ^6^ Pharmacological research has made tremendous strides recently, resulting in the discovery of mushroom-based medications.^7^ Two such drugs, PSK (polysaccharide-K)—developed from *Trametes versicolor* (Turkey tail) and Lentinan from *Lentinula edodes*—have revealed noteworthy promise in cancer intervention.^8^^,^^9^ Mushrooms are promising agents for cancer treatment owing to their unique bioactive compounds, such as polysaccharides, triterpenoids, and lectins.^10^, ^11^, ^12^ These molecules exhibit anti-cancer activity by modulating signaling pathways involved in cancer cell growth, apoptosis, and invasion, underscoring their potential as novel therapeutic agents.^13^

Cancer, a multifaceted disease, remains a significant global health concern, causing around 10 million deaths in 2020, as per the World Health Organization.^14^ Globally, cancer types responsible for the highest mortality rate comprise lung, colorectal, liver, stomach, and breast cancers, followed by prostate, cervical, esophageal, bladder, pancreatic, and head and neck cancers, along with non-Hodgkin lymphoma and leukemia.^15^^,^^16^ Conventional cancer treatments—chemotherapy, radiation, and surgery—often achieve efficacy but are frequently associated with immunosuppression, fatigue, and organ toxicity.^17^^,^^18^ These drawbacks highlight the critical need for alternative therapeutic approaches.

Natural products have gained increasing attention in oncology due to their diverse bioactive constituents and relatively low toxicity.^19^ Of these, mushrooms present a promising option, serving as a reservoir of distinct bioactive molecules that may target diverse pathways involved in cancer progression.^20^ Mushrooms exhibit anti-cancer activity through apoptosis induction, immune modulation, and anti-angiogenesis in cancer cells.^21^, ^22^, ^23^, ^24^ Their potential resides in their capacity to enhance the effectiveness of traditional therapeutic approaches while decreasing their side effects, making them an appealing target for research.^24^

Autophagy plays a dual role in cancer, acting as a tumor suppressor by removing harmful cellular components and as a tumor promoter by supporting cancer cell survival under stress.^25^, ^26^, ^27^, ^28^ Targeting autophagy offers a promising therapeutic strategy, either by inducing it to trigger cancer cell death or inhibiting it to enhance treatment sensitivity.^29^, ^30^, ^31^, ^32^ Mushrooms offer a rich, natural source of autophagy modulators with significant potential to exploit this dual role for therapeutic benefits in cancer.^33^ They play a significant role in this context, as certain bioactive compounds found in mushrooms modulate autophagy. Mushrooms are notable for their bioactive compounds that regulate autophagy: for instance, triterpenoids induce autophagy-mediated cancer cell death,^34^ whereas cordycepin inhibits autophagy by suppressing the AMP-activated protein kinase (AMPK)/mammalian target of rapamycin (mTOR)/uncoordinated-51 (unc-51)-like autophagy activating kinase 1 (ULK1) pathway and lysosomal function, thereby enhancing the efficacy of conventional therapies by sensitizing cancer cells.^35^

The intrinsic ability of mushroom-derived bioactives to selectively modulate autophagy pathways presents a unique therapeutic opportunity, leveraging the natural safety profile of these compounds to enhance the efficacy and synergy of cancer treatments. This review examines mushrooms as autophagy modulators in cancer therapy, highlighting their bioactive compounds, mechanisms, and potential to enhance treatment efficacy while reducing side effects. It explores new research avenues and therapeutic applications, positioning mushroom-derived compounds as promising agents for safer, more effective cancer treatments.

## Brief overview of autophagy and its mechanism

**Figure 1:**
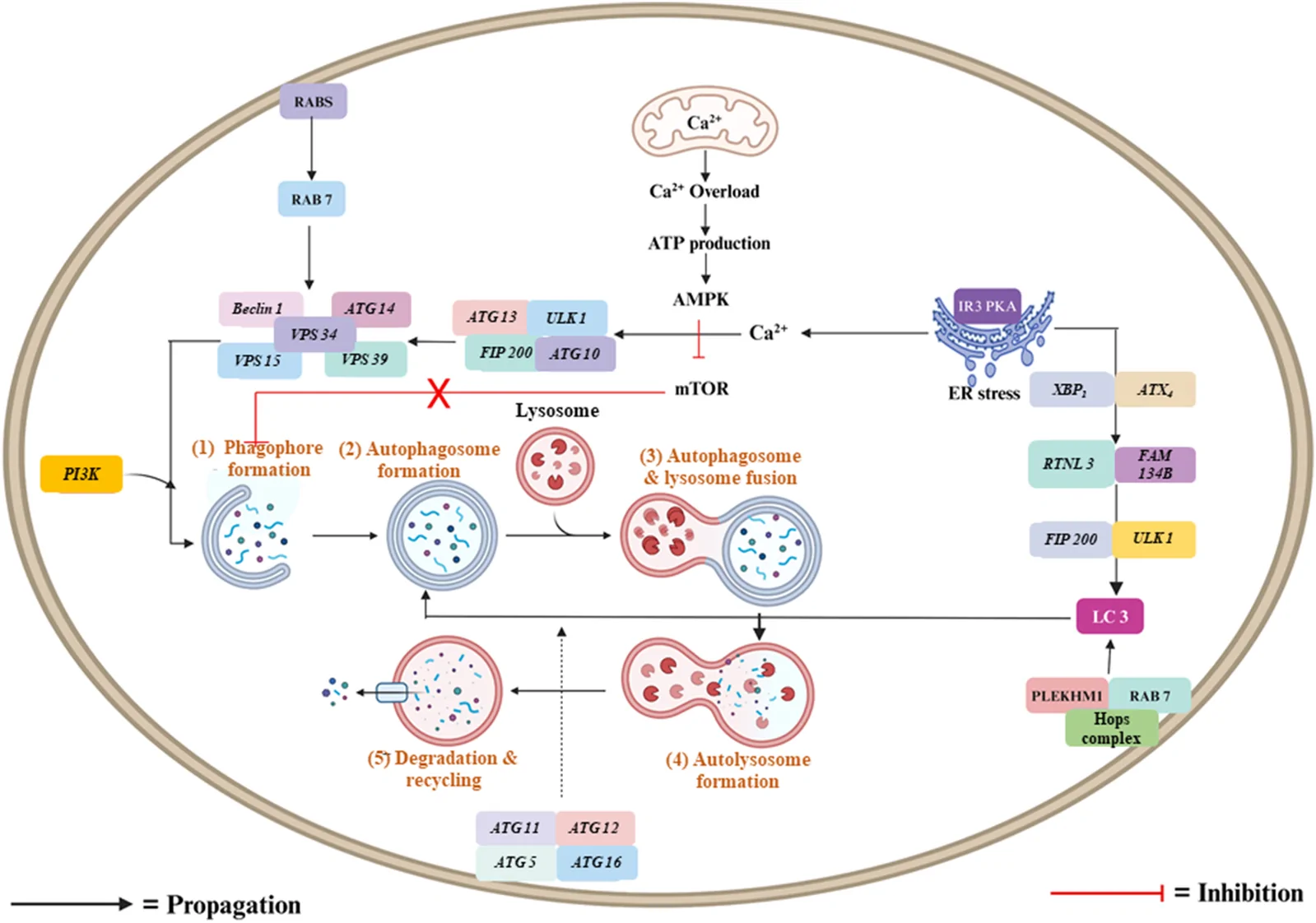
Autophagy signaling pathway and regulatory mechanisms. Autophagy begins with the formation of the phagophore (1), which elongates and matures into a double-membraned autophagosome (2). The autophagosome subsequently fuses with lysosomes to generate an autolysosome (3), where the sequestered cellular components are degraded and recycled (4). The process is orchestrated by key regulatory proteins, including Beclin-1, ATG proteins, VPS complexes, and LC3, and modulated by upstream signals such as PI3K activation, ER stress, calcium flux, and mTOR inhibition. AMPK promotes autophagy through mTOR suppression, whereas Rab family proteins facilitate vesicle trafficking. AMPK: AMP-activated protein kinase; ATG: Autophagy-related proteins; ATP: Adenosine triphosphate; LC3: Microtubule-associated protein 1A/1B-light chain 3; mTOR: Mechanistic target of rapamycin kinase; PINK 1: PTEN-induced kinase 1; RABs: Ras-associated binding proteins; RAB7: Ras-associated binding protein 7; ULK1: Unc-51-like autophagy-activating kinase 1; VPS: Vacuolar protein sorting.

Autophagy, a conserved recycling mechanism in all eukaryotes, has three forms in mammals involving macroautophagy, microautophagy, and chaperone-mediated autophagy, all culminating in lysosomal degradation.^36^^,^^37^ Macroautophagy, the most studied form of autophagy, is a basal process that intensifies under stress conditions like nutrient or energy deprivation. It involves the degradation of cytoplasmic components into metabolites used for energy production or biosynthesis, thereby supporting cell survival.^37^^,^^38^ Under typical growth conditions, macroautophagy helps maintain cells by destroying damaged or unnecessary organelles. Therefore, the primary role of macroautophagy is cytoprotection; however, excessive self-degradation can be harmful. This dynamic process involves autophagosome formation, fusion with lysosomes, and degradation of autophagosomal contents by lysosomal hydrolases.^39^ The recognition of homologs in higher eukaryotes after the elucidation of autophagy-related genes (*ATG*) in yeast contributed to our molecular understanding of autophagy. A subset of these ATG proteins, referred to as the “core” molecular machinery, is necessary for the formation of autophagosomes.^39^^,^^40^ The core ATG proteins are categorized into four functional subgroups: (1) the ATG1/ULK kinase complex, (2) two ubiquitin-like conjugation systems involving ATG12 and ATG8/LC3 (microtubule-associated protein 1 light chain 3), (3) the class III phosphatidylinositol 3-kinase (PI3K)/vacuolar protein sorting 34 (Vps34) complex I, and (4) two transmembrane proteins, ATG9/m ATG9 and vacuole membrane protein 1 (VMP1), along with regulatory partners such as ATG18/WD repeat domain phosphoinositide-interacting protein 1 (WIPI1) that facilitate ATG9 trafficking. The ULK complex, consisting *of ATG13*, *ULK1/2*, focal adhesion kinase (FAK) family-interacting protein of 200 kDa (FIP200), and *ATG101*, is essential for the initiation of autophagy.^39^^,^^41^ By determining the level of adenosine monophosphate (AMP) and adenosine triphosphate (ATP), AMPK functions as an energy-sensing kinase that promote autophagy.^42^^,^^43^ Additionally, Ca^2+^ signaling and endoplasmic reticulum (ER) stress play crucial roles in regulating autophagy by influencing AMPK and Beclin-1 activity.^44^ The Beclin-1 complex, which includes Beclin-1, *Vps34*, *Vps15*, and *ATG14*, is essential for initiating phagophore formation through generating phosphatidylinositol 3-phosphate (PI3P), a key lipid that recruits proteins necessary for vesicle expansion.^45^ During nutrient starvation or energy depletion, the AMPK is induced, triggering the ULK1/2 complex.^46^ This complex further stimulates the Beclin-1 complex, inducing PI3P-mediated phagophore, which is nucleated thereafter. Following maturation, autophagosomes fuse with lysosomes to form autolysosomes, where lysosomal hydrolases degrade the cargo. This fusion is regulated by LC3, Ras-related protein Rab-7a (*RAB7*), pleckstrin homology domain containing family M member 1(*PLEKHM1*), and the homotypic fusion and vacuole protein sorting (HOPS) complex. The resulting amino acids, fatty acids, and nucleotides are recycled for biosynthesis and energy production.^47^^,^^48^ Moreover, autophagy is induced when activated AMPK phosphorylates the tuberous sclerosis complex (TSC) and reduces the mTOR activity.^49^^,^^50^ As mTOR prevents autophagy, mTOR suppressors have been designed to induce autophagy. Moreover, *ATG4* cleaves the precursor proLC3 to form LC3-I, whereas *ATG7, ATG3*, and the ATG12–ATG5–ATG16 complex conjugate the phosphatidyl-ethanolamine (PE) phospholipid to form LC3-II. The ATG12–ATG5–ATG16L1 complex, the class III phosphatidylinositol 3-kinase (PtdIns3K) complex, LC3-II, and *ATG9* are devoted to the phagophore.^51^, ^52^, ^53^ Subsequently, the elongating phagophore engulfs cytoplasmic material to form a double-membraned autophagosome, with LC3-II dissociating from its outer membrane. Fusion with a lysosome creates an autolysosome, where contents are degraded and recycled back into the cytoplasm for reuse [Figure 1].^54^

In summary, upstream regulators such as AMPK, mTOR, calcium signaling, and ER stress act as metabolic and stress sensors that determine autophagy initiation. These signals act on ULK1 and Beclin-1 complexes. AMPK activates ULK1 and inhibits mTOR, lifting its suppression. The Beclin-1–Vps34–ATG14 complex produces PI3P, whereas A*TG13*, FIP200, and *VPS15* serve as scaffolds in the cascade.^51^, ^52^, ^53^

Following upstream activation, ATG12–ATG5–ATG16L1 conjugation drives isolation membrane elongation, whereas LC3-II marks autophagosomes for cargo loading and fusion. *RAB7, PLEKHM1*, and the HOPS complex mediate autophagosome–lysosome fusion, ensuring autophagic flux and degradation by lysosomal hydrolases. These mechanisms highlight potential therapeutic targets in autophagy-related diseases.^47^^,^^48^

## Autophagy’s Dilemma: “tumor suppressor or supporter?”

**Figure 2:**
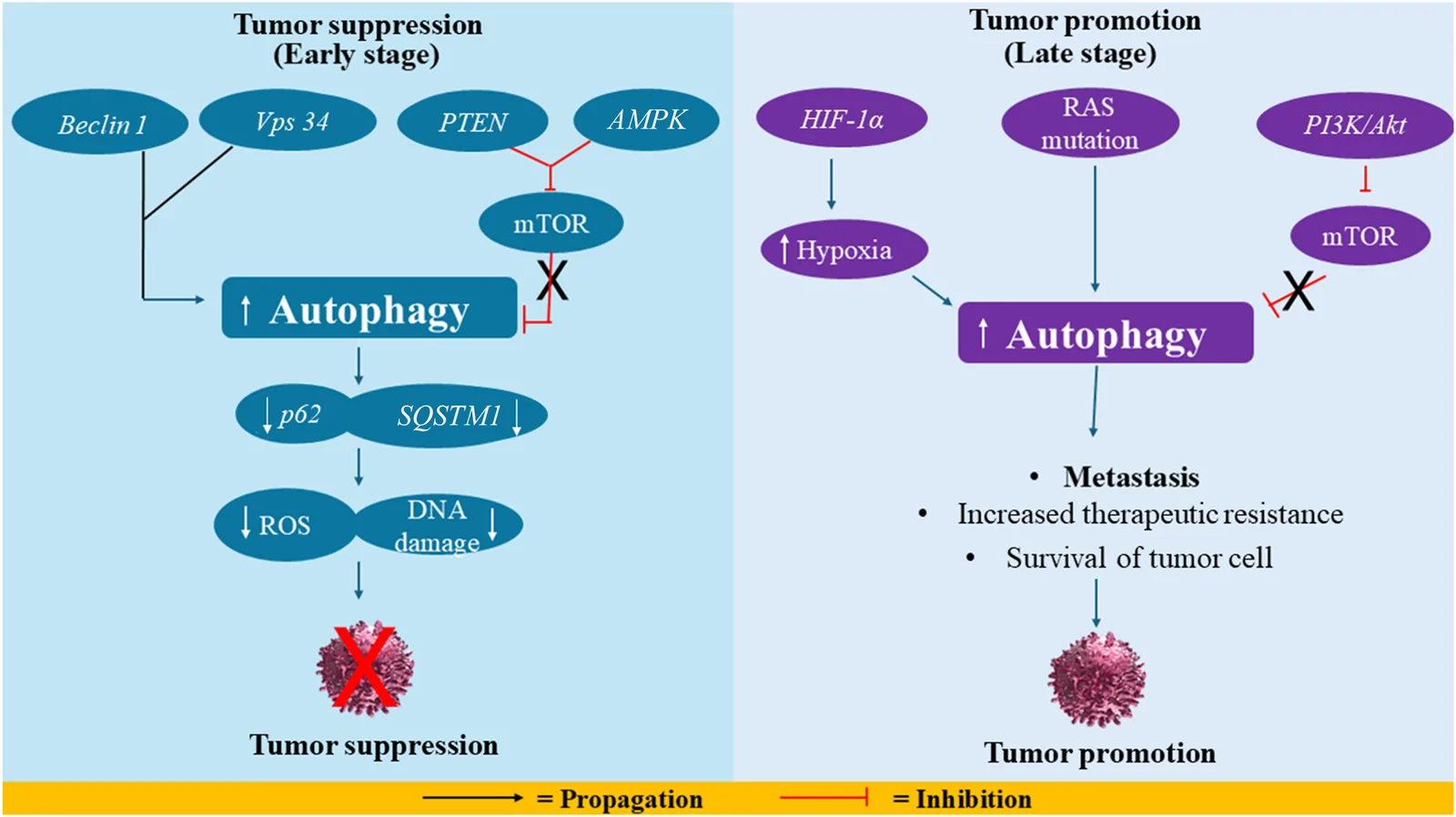
Schematic representation of the dual role of autophagy in cancer. In the early stages, upregulation of Beclin-1 and *Vps34* activates autophagy, reducing ROS accumulation and limiting DNA damage, thereby suppressing tumor initiation. In advanced stages, factors such as *HIF-1α* activation, *RAS* mutations, and mTOR inhibition further enhance autophagy, promoting metastasis, therapeutic resistance, and tumor progression. *Akt*: Protein kinase B; *HIF-1α*: Hypoxia-inducible factor 1-alpha; AMPK: AMP-activated protein kinase; mTOR: Mechanistic target of rapamycin kinase; *PI3K*: Phosphoinositide 3-kinase; *PTEN*: Phosphatase and tensin homolog; *RAS*: Rat sarcoma oncogene; ROS: Reactive oxygen species; p62/SQSTM1: Sequestosome 1; *Vps34*: Vacuolar protein sorting 34.

Autophagy, a cellular recycling mechanism, exerts tumor-suppressive or cancer-promoting effects depending on cancer type, stage, and genetic background [Figure 2].^55^ In early tumorigenesis, autophagy maintains cellular quality by removing damaged organelles and proteins, preserving genomic stability, activating tumor suppressors, regulating PI3K/Akt signaling, and preventing oxidative stress to inhibit cancer development.^56^ Later, tumor cells exploit autophagy to survive hypoxia, nutrient shortage, and chemotherapy, supporting their growth and metabolic needs.^57^

Ganoderic acid DM (GA-DM) from *G. lucidum* illustrates autophagy’s dual role in prostate cancer. It suppresses tumors by upregulating Beclin-1 and *ATG5*, inducing apoptosis via B-cell lymphoma 2 (Bcl-2)/Bcl-2-associated X (Bax) and caspase-3, and boosting ER stress and immune response. However, under prolonged stress, GA-DM can shift autophagy to a survival mechanism, promoting cancer cell protection and highlighting its paradoxical effects in cancer therapy.^58^

### Tumor suppression: role of autophagy in preventing cancer progression

Autophagy suppresses tumor development by reducing cell proliferation, relieving cellular stress, and preserving genomic stability through the clearance of damaged components.^59^ Key genes like *Beclin*-1 play a vital role; its monoallelic loss leads to spontaneous tumors in mice and is frequently observed in human breast, ovarian, and prostate cancers.^60^ Beclin-1 activates autophagy via *Vps34* interaction, reinforcing its tumor-suppressive function.^61^ Conversely, impaired autophagy is linked to the progression of several cancers, including lung, liver, colorectal, and gastric cancer.^62^^,^^63^

Defective autophagy leads to the accumulation of *p62/sequestosome 1 (SQSTM1*) aggregates, damaged mitochondria, and misfolded proteins, causing increased reactive oxygen species (ROS) production. This triggers DNA damage and genomic instability, driving tumor initiation and progression. Moreover, impaired autophagy disrupts endoplasmic reticulum (ER) homeostasis, causing persistent ER stress from accumulated unfolded and misfolded proteins.^64^^,^^65^ Chronic unfolded protein response (UPR) activation without functional autophagy intensifies cellular stress and promotes tumorigenesis. Functional autophagy mitigates ER stress by degrading protein aggregates, restoring ER function, and suppressing tumor-promoting signals. Calcium (Ca^2+^) dynamics are closely tied to autophagy, as the ER is a major Ca^2+^ reservoir. Impaired autophagy disrupts Ca^2+^ signaling, causing abnormal cytoplasmic release that activates pro-tumorigenic pathways and elevates oxidative stress, promoting cancer progression. Restoring autophagy rebalances Ca^2+^ levels, reducing oxidative stress and cellular damage.^66^ Additionally, reducing *p62/SQSTM1* levels in autophagy-deficient cells lowers ROS production and DNA damage. In lung cancer models, *p62/SQSTM1*-deficient animals resist Ras-induced tumors, unlike wild-type controls. Accumulation *of p62/SQSTM1* and increased ROS thus links impaired autophagy to cancer progression.^67^

Furthermore, other autophagy-related genes, such as *ATG5, ATG7,* and *ATG4C*, have vital tumor-suppressive roles in liver, pancreatic, gastric, and colorectal cancers. Mice deficient in *ATG5* or *ATG7* develop multiple liver tumors from elevated oxidative stress and mitochondrial damage, highlighting the essential roles of these genes in cellular homeostasis and tumor suppression. Although *ATG4C* contributes to autophagosome formation, its absence has a less pronounced effect on tumorigenesis compared to *ATG5* and *ATG7*, likely due to the presence of compensatory isoforms.^68^ Additionally, dysregulation of signaling pathways like PI3K/Akt underscores autophagy’s role in tumor suppression. In gliomas and breast, lung, and prostate cancers, PI3K/Akt overactivation suppresses autophagy and promotes tumor growth. PTEN loss or Akt activation reduces autophagy, causing p62 accumulation, increased oxidative stress, and poor prognosis. For instance, in glioma cells, *PTEN* loss or Akt activation diminishes autophagic activity, thereby exacerbating tumorigenesis by increasing cellular stress and genomic instability.^69^

Autophagy also plays a role in blocking tumor growth by reducing necrosis and chronic inflammation, which are associated with cancer progression in melanoma, colorectal, pancreatic, and gastric cancers. Autophagy clears damaged cells, preventing the release of pro-inflammatory factors like high mobility group box 1 (*HMGB1*) that promote a tumor-supportive microenvironment. By reducing necrosis and inflammation, autophagy limits *HMGB1* production, thereby inhibiting tumor growth and spread.^59^, ^60^, ^61^

### Tumor promotion: how autophagy enables cancer progression

Autophagy acts as a tumor suppressor in early cancer stages but shifts to a pro-survival mechanism in advanced tumors.^70^ As malignancies grow rapidly and face metabolic stress from poor vascularization and inflammation, autophagy helps cancer cells adapt by supporting survival under nutrient and oxygen deprivation, thereby sustaining tumor growth and its microenvironment.^71^^,^^72^

A key mediator of the inositol-requiring enzyme 1 alpha–X-box binding protein 1 (IRE1α–XBP1) axis promotes autophagy-mediated tumor survival, with IRE1α enhancing tumor cell viability through regulated IRE1-dependent decay (RIDD) of target mRNAs. In this process, IRE1α splices XBP1 mRNA, which induces transcription of pro-survival autophagy genes.^65^ This also activates tribbles pseudokinase 3 (TRB3), further enhancing autophagy to protect tumor cells from harsh microenvironments, contributing to therapy resistance by enabling cancer cells to evade apoptosis post-chemotherapy and triggers autophagic cell death though excessive activation of ATF4 pathway.^73^

The protein kinase RNA-like endoplasmic reticulum kinase (PERK)–eukaryotic initiation factor 2 alpha (eIF2α)–activating transcription factor 4 (ATF4) pathway is a key regulator of autophagy-driven tumor promotion. It enhances autophagy under stress by inducing genes that support nutrient recycling and energy balance, helping cancer cells survive and resist apoptosis. In solid tumors, this adaptive response contributes to treatment resistance, though excessive activation can trigger autophagic cell death.^74^

The activating transcription factor 6 (ATF6) pathway promotes tumor progression by inducing autophagy-related genes, supporting cancer cell survival through proteostasis maintenance and relief of ER stress.^75^ Additionally, site-1 protease (S1P) and site-2 protease (S2P)-mediated cleavage of *ATF6* in the Golgi apparatus enhances autophagic flux, enabling tumor cells to evade stress-induced apoptosis—an effect particularly critical in aggressive cancers reliant on ER homeostasis for survival.^76^

Calcium signaling, which plays a tumor-suppressive role under excessive stress, can support tumor survival under moderate conditions.^77^ Activation of calcium/calmodulin-dependent protein kinase (CaMK) and c-Jun N-terminal kinase (JNK), downstream of ER calcium release, enhances autophagy to promote cancer cell adaptation over apoptosis, enabling stress resistance and tumor survival.^78^

The pro-survival role of autophagy poses challenges in cancer therapy, as tumors exploit it to resist treatment and recover from damage. Inhibitors like chloroquine and hydroxychloroquine enhance therapy by blocking nutrient recycling.^79^^,^^80^ However, autophagy inhibition can trigger necrosis and inflammation, potentially fostering a tumor-supportive environment.

## Impact of mushrooms as autophagy modulators in different types of cancer

Recent studies have shown that extracts from various mushrooms [Table 1] and their bioactive compounds [Table 2] influence cancer cells *in vitro* and *in vivo* by modulating autophagy. Moreover, comparative studies of the mushrooms with translational potential are depicted in Table 3.

**Table 1:** Mushrooms with autophagy-modulating capacity for chemotherapeutics and chemoprevention.

Types of cancer	Scientific Name	Family and common name	Types of extract	Test model used	Mechanism	Role in cancer	Ref.
Colorectal Cancer	*Ganoderma lucidum*	Family: *Ganodermataceae* Common name: Reishi	Triterpene extract	*In vitro*: HT-29 colorectal cell lines *In vivo*: Xenograft mice model	p38 inhibition in HT-29 colorectal cells induces autophagy by increasing Beclin-1 and LC3 expression and promoting autophagosome formation.	*In vitro*:↓ (decrease) cell proliferation *In vivo*: ↓tumor growth	96
			Fruiting body extract of *G. lucidum*	*In vitro*: HCT 116 cells *In vivo*: HCT 116 xenografted mice model	In HCT 116 murine models, autophagy is induced by increasing Beclin-1, the LC3B/LC3A ratio, *ATG5*, and total mTOR, while reducing p-mTOR levels.	*In vitro*:↓cell proliferation *In vivo*: ↓tumor growth	97
			*G.lucidum* polysaccharide	*In vitro*: HT-29 and HCT 116 cells *In vivo*: Male BALB/c nude mice injected with HT-29 cells	GLP derived from *G.lucidum* spores using hot water extraction stimulated autophagy by enhancing the synthesis of p62 and LC3-II proteins and activating the mitogen-activated protein kinase/Extracellular signal-regulated kinase (MAPK/ERK) pathway.	*In vitro*: apoptosis-mediated, cell death; cytotoxicity *In vivo*: ↓tumor growth	95
	*Antrodia cinnamomea*	Family: *Polyporaceae* Common name: Niu Chang-Zhi’ or ‘Niu Chang-Gu’ in Chinese	*A. cinnamomea* extracts	*In vitro*: HCT 116, HT-29, SW480, Caco-2 and, Colo205 colorectal cancer cells *In vivo:* HCT 116 cells in athymic mice	An ethanolic extract induces autophagy in HCT 116 cells by upregulating *CHOP* and *TRIB3*, which frees ULK1 to boost autophagosome formation and suppresses mTOR through Akt. *ACF2* promotes autophagy, shown by increased LC3-II levels.	*In vitro*: cytotoxicity *In vivo*: ↓tumor volume	99
	*Antrodia salmonea*	Family: *Polyporaceae* Common name: Guiseniuzhangzhi	Fermented culture broth of *Antrodia salmonea*	*In vitro*: SW620 cells	Induced cytoprotective autophagy by suppressing Akt/mTOR signaling, NFκB, and β-catenin expression, while accumulating LC3-II, activating *p62/SQSTM1*, inactivating *ATG4B*, forming acidic vesicular organelles (AVOs), and disrupting the beclin-1/Bcl-2 complex.	*In vitro*: cytoprotective effect	100
	*Pleurotus tuber*	Family: *Pleurotaceae* Common name: Oyster mushroom	Selenium nanoparticle-conjugated extract from Pleurotus tuber-regium	*In vitro*: HCT 116 cells	Triggered autophagy by elevating the levels of LC3-II and beclin 1, while the expression of *p62/SQSTM1* was decreased.	*In vitro*: autophagic cell death	101
	*Phellinus linteus(*Hymenochaetaceae)*, Grifola frondosa(*Aphyllopherales)*, Hericium erinaceum(Hericiaceae), Lentinula edodes(*Marasmiaceae)*, Sparassis crispa(*Sparassidaceae)*, Trametes versicolor(*Polyporaceae), *Cordyceps Militaris* (Cordycipitaceae) and *Panax ginseng* (Araliaceae)		Amex7 extract	*In vitro*: HT-29 cell line	Amex7 was found to elevate p62 and LC3A/B-II expression levels, thereby promoting autophagy in HT-29 human colorectal cancer cells.	*In vitro*: cell cycle arrest and cell death *In vivo*: ↓tumor growth	102
Gastric cancer	*G.lucidum*	Family: Ganodermataceae, common name: Reishi, Varnished conk, or ling Chih,	Methanolic *G. lucidum* extract	*In vitro*: Human GC AGS cell line	Induction of autophagy by increasing LC3-II and autophagosome monodansylcadaverine tagging	*In vitro*: ↓cell proliferation	27
			Methanolic *G. lucidum* extract	*In vitro*: Human GC AGS cell line	Autophagy is induced by enhancing the formation of autophagosomes, elevating cellular levels of LC3-II, and reducing cellular levels of p62	*In vitro*: ↓cell growth	107
			Cold methanolic *G.lucidum* extract	*In vitro*: Human GC AGS cell line	Induction of autophagy through increasing the formation of autophagosomes	*In vitro*: ↓cell growth	108
	*Lactarius deterrimus*	Family: *Russulaceae* Common name: False Saffron Milkcap	Ethanol extract of *Lactarius deterrimus*	*In vitro*: Human GC AGS cells (ATCC, CRL-1739)	Induction of autophagy through cytoskeleton rearrangements	*In vitro*: cytotoxic and anti-invasive effects	110
Breast cancer	*Inonotus obliquus* (enriched in inotodiol and trametenolic acid)	Family: *Hymenochaetaceae* Common name: Chaga	Ethanol extract of Chaga mushroom	*In vitro*: MDA-MB-231, MDA-MB-468, and MCF-7 *In vivo*: 4T1 tumor-bearing BALB/c mice	Autophagy is induced in 4T1 mouse breast cancer cells by activating AMPK and inhibiting mTOR signaling pathways	*In vitro*: ↓cell proliferation *In vivo*: ↓tumor growth	120
	*Trametes robiniophila*	Family: *Polyporaceae* Common name: Huaier	Huaier extract	*In vitro:* MDA-MB-231, MDA-MB-468, and MCF-7 breast cancer cells *In vivo*: BALB/c nu/nu mice	Autophagy is induced through suppression of the mTOR/S6K pathway in breast cancer cell lines MDA-MB-231, MDA-MB-468, and MCF-7	*In vitro*: ↓cell viability and cytotoxicity *In vivo*: ↓tumor growth	121
	*Antrodia salmonea* *Antrodia salmonea*	Family: *Fomitopsidaceae*Common name: N/A Family: *Fomitopsidaceae*Common name: N/A	Fermented *Antrodia salmonea* culture broth Fermented *Antrodia salmonea* culture broth	*In vitro*: MDA-MB-231 *In vivo:* MDA-MB-231 xenografted nude mice *In vitro*: MDA-MB-231 *In vivo:* MDA-MB-231 xenografted nude mice	Autophagy is induced in MDA-MB-231 cells, yielding a notable accumulation of lipidated LC3, the formation of GFP-LC3 punctate patterns, and the production of AVOs. Autophagy is induced in breast cancer cells through increased accumulation of LC3-II and upregulation of *ATG7*, causing cell death and mitochondrial dysfunction	*In vitro*: ↓cell viability *In vivo*: ↓tumor growth	123,176
	*Antrodia cinnamomea*	Family: *Polyporaceae*Common name: N/A	Ethanolic extracts of *Antrodia cinnamomea*	*In vitro:* T47D cells	Autophagy is induced by triggering endoplasmic reticulum stress via *IRE1* activation in T47D cells, leading to the production of the anticancer protein CHOP and activation of acetyl-histones H3 and H4 through HDAC inhibition	*In vitro*: ↓cell growth	177
	*G.lucidum*	Family: Ganodermataceae Common name: Reishi	DMSO extract of *G. lucidum*	*In vitro*: MCF-7 and MDA-MB-231	Autophagy is initiated in MCF-7 and MDA-MB-231 cells, resulting in increased expression levels of Beclin-1, LC3, and p62	*In vitro*: ↓cell proliferation and cell cycle arrest	124
	*Phellinus linteus*	Family: *Hymenochaetaceae* Common name: Meshimakobu	5-Fluorouracil with ethanol extract from the fruiting bodies of *Phellinus linteus,*	*In vitro*: Human triple-negative breast cancer (MDA-MB-231) cells	Autophagy is induced in MDA-MB-231 triple-negative breast cancer cells by increasing LC3-I to LC3-II conversion, forming acidic vesicular organelles (AVOs) and revealing double-membraned vacuoles at the ultrastructural level, especially when combined with a low dose of 5-FU	*In vitro*: ↓cell proliferation and autophagic cell death	125
Liver cancer	*Hypsizygus marmoreus*	Family: *Lyophyllaceae* Common name: Jade Mushroom/Zhengjigu	Extract from fruiting bodies of *Hypsizygus marmoreus*	*In vitro*: Hep 3B cells	Induction of autophagy by converting LC3-I to LC3-II and by increasing levels of p62 and p62p (S403)	*In vitro*: autophagic cell death	134
	*Inonotus baumii*	Family: *Hymenochaetaceae* Common name: Sanghuang	*Inonotus baumii* extract,	*In vitro*: SMMC-7721 *In vivo*: BALB/c nude mice	Induction of autophagy via the AMPK/mTOR/ULK1 pathways	*In vitro*: autophagic cell death *In vivo*: ↓tumor growth	135
	*G.lucidum*	Family: Ganodermataceae Common name: Reishi/Lingzhi	extract of *G. lucidum* spore powder	*In vitro*: HepG2 and Huh6 human HB cell lines *In vivo*: BALB/c mice	Autophagy was induced by an increase in the LC3-II/LC3-I ratio and a decrease in p62, both of which indicate autophagy	*In vitro*: ↓cell migration. *In vivo*: ↓tumor growth	136
	*Grifola frondosa*	Family: *Grifolaceae* Common name: Maitake	Cold-water extract of *Grifola frondosa* (GFW) and its active fraction (GFW-GF)	*In vitro*: Human HCC Hep3B, HA22T, and Huh7 cells *In vivo*: Five-week-old male BALB/c athymic nude mice	Induction of autophagy by inhibiting PI3K and JNK pathways	*In vitro*: ↓cell proliferation *In vivo*: ↓tumor growth	137
Ovarian cancer	*Antrodia salmonea*	Family: *Fomitopsidaceae*	Aqueous hyphae extracts of *Antrodia salmonea*	*In vitro*: SKOV-3 and A2780 ovarian cancer cell lines	Autophagy is induced by increased levels of LC3-II, GFP-LC3 puncta, and AVO formation, alongside the activation of *p62/SQSTM1*, suppression of *ATG4B*, upregulation of *ATG7*, and disruption of the beclin-1/Bcl-2 complex	*In vitro*: ROS-mediated autophagic cell death	146
Cervical cancer	*Sanghuangporus baumii*	Family: *Hymenochaetaceae*Common name: N/A	Aqueous extract of *Sanghuangporus baumii*	*In vivo*: U14 cervical cancer cell lines were implanted in female Kunming mouse models	Autophagy is induced by upregulating the expression of key genes *GABARAP, VMP1, VAMP8*, and *STX17* while disrupting glucose uptake and utilization in tumors	*In vivo*: ↓tumor growth	153
	*Lenzites betulina*	Family: *Polyporaceae* Common name: gilled polypore	The ethanolic extract of *Lenzites betulina*	*In vitro*: HeLa cell *In vivo*: HeLa-implanted mice	Autophagy is induced in HeLa cells through the formation of autophagic vacuoles Notably, the interaction of 4′’-hydroxy-6-methoxyaurone with P-glycoprotein diminishes the cancer cells’ capacity to develop drug resistance	*In vitro*: cell cycle arrest, and inhibition of cell invasion *In vivo*: ↓tumor growth	154
	*Pleurotus ostreatus* *Pleurotus eryngii*	Family: *Pleurotaceae* Common name: oyster mushroom Family: *Pleurotaceae* Common name: king oyster mushroom	Water-soluble extracts from the mycelium of *Pleurotus ostreatus* and *Pleurotus eryngii*	*In vitro*: SiHa cells	Induction of autophagy in SiHa cells by the ER stress-mitochondrial pathway	*In vitro*: autophagic cell death	155
Skin cancer	*Trametes versicolor*	Family: *Polyporaceae* Common name: Coriolus versicolor	An ethanolic extract from the fruiting body and mycelium of *Trametes versicolor*	*In vitro:* SK-MEL-5	Induction of autophagy in the SK-MEL-5 human melanoma cell line by upregulating the autophagy marker LC3-II	*In vitro*: programmed cell death and inhibition of cell migration	159
	*Cordyceps militaris* (cordyceps medium-loaded nanoparticles)	Family: *Cordycipitaceae* Common name: Orange caterpillar fungus	Nanoencapsulated cordyceps extract	*In vitro*: Human dermal fibroblasts (HDFs)	Promotion of autophagy to enhance the regeneration of human dermal fibroblasts (HDFs) and suppression of oxidative stress generated by H_2_O_2_	*In vitro*: cell regeneration and stress protection	161
Leukemia	*Ganoderma tsugae*	Family: Ganodermataceae Common name: Reishi	*Ganoderma tsugae’s* ethanolic extracts	*In vitro*: K562 cells	Activation of cytoprotective autophagy in leukemia K562 cells by upregulating LC3-II, dysregulating beclin-1/Bcl-2, promoting the production of acidic vesicular organelles, and activating *p62/SQSTM1*	*In vitro*: autophagic cell survival.	165
Head and neck cancer	*Antrodia salmonea*	Family: *Polyporaceae* Common Name: Niuzhangzhi	Submerged fermented broth *of A. salmonea*	*In vitro*: HNSCC Twist-overexpressing (OECM-1 and FaDu-Twist) cells *In vivo*: OECM-1-xenografted nude mice	Autophagy activation, marked by LC3-I/II accumulation, AVO formation, and *p62/SQSTM1* expression, contributes to apoptosis and effectively reduces tumor volume and weight in OECM-1 xenograft models *in vivo*	*In vitro*: ↓cell viability *In vitro*: ↓tumor volume and weight	171

Note: Mechanisms described as “increased/decreased expression” or “up/downregulation” refer to protein levels unless stated otherwise. Transcriptional changes are explicitly noted. Akt: Protein kinase B; AMPK: AMP-activated protein kinase; ATG: Autophagy-related protein; ATP: Adenosine triphosphate; CHOP: C/EBP homologous protein; ER: Endoplasmic reticulum; GLE: *G. lucidum* extract; GLP: *G. lucidum* polysaccharide; GLR: *G. lucidum* ribonuclease; HIF-1α: Hypoxia-inducible factor 1-alpha; LC3-I/II: Microtubule-associated protein 1A/1B-light chain 3, form I/II; MAPK/ERK: Mitogen-activated protein kinase/extracellular signal-regulated kinase; mTOR: Mechanistic target of rapamycin; PINK: PTEN-induced kinase 1; PI3K: Phosphoinositide 3-kinase; PTEN: Phosphatase and tensin homolog; RABs: Ras-associated binding proteins; RAB7: Ras-associated binding protein 7; RAS: Rat sarcoma oncogene; ROS: Reactive oxygen species; SQSTM1: Sequestosome 1; TRB3: Tribbles pseudokinase 3; ULK1: Unc-51-like autophagy-activating kinase 1; VPS: Vacuolar protein sorting; -: Not available..

**Table 2:** Bioactive substances from the autophagy of mushrooms effective in cancer.

Types of cancer	Compound name	Compound types	Source	Test model used	Findings	Autophagy modulation mechanism	Role in cancer	Ref.
Lung cancer	Ganoderic acid DM	Triterpenoid	*G. lucidum*	*In vitro*: A549 and NCI-H460 cell lines	Transmission electron microscopy revealed numerous autophagolysosomes and autophagic bodies in A549 and NCI–H460 cells after 48 h treatment with 10 μmol/L GA-DM, while no such structures were observed in control cells (*P* < 0.05).	Autophagic flux is induced and autophagy is activated by inhibiting the PI3K/Akt/mTOR pathway in A549 and NCI-H460 cell lines.	*In vitro*: ↓ (decrease) cell proliferation and induction of apoptosis	82
	Gibbosic acid H (GaH)	Tetracyclic diterpenoid acid	*Ganoderma* (Ganodermataceae) species	*In vitro*: A549 and H1299 cell line	GaH treatment (10 μmol/L, 24 h) in non-small cell lung cancer (NSCLC) cells (A549 and H1299) reduced p62 and increased LC3B, indicating enhanced autophagy. This effect, mediated by AMPK activation, suggests GaH’s antiproliferative activity is partly due to autophagy induction.	Induction of autophagy via activated adenosine monophosphate-activated protein kinase (AMPK) and increasing expression of p53, beclin-1, and light chain 3 beta (LC3B).	*In vitro*: ↓cell proliferation	83
	Antrodin C	Maleimide	*Niuzhangzhi*	*In vitro*: SPCA-1 cell line	-	Activation of autophagy in SPCA-1 cells by upregulating light chain 3 beta (LC3-II) expression, a marker of autophagosome formation, and suppressing the Akt-mTOR and AMPK pathways.	*In vitro*: ↓cell proliferation	84
				*In vitro*: A549 cancer cell line	*-*	Stimulation of autophagy, which inhibited the growth of the A549 lung cancer cell line in a cytoprotective manner.	*In vitro*: inhibition of apoptosis	84
	Cordycepin	Nucleoside analog	*Cordyceps militaris*	*In vitro*: H1792, H1299, H460, H1-57, and A549 cell lines	Cordycepin-induced autophagy facilitates apoptosis in H460 and H1299 cell lines (*P* < 0.05).	Inhibition of the mTOR pathway induces autophagy in human non-small cell lung cancer cell lines (H1792, H1299, H460, H157, and A549), leading to the autophagy-mediated degradation of c-FLIPL and resulting in cell death.	*In vitro*: ↓cell viability	85
	Astragurkurol	Triterpenoid	*Astraeus hygrometricus*	*In vitro*: A549 cell line *Invivo*: *Ex ovo* xenograft mode	Astrakurkurol (61.8 μmol/L) dose-dependently reduced the phosphorylation of PI3K and mTOR proteins in A549 cells (*P* < 0.001).	Induction of autophagy in A549 lung cancer cells by promoting the proliferation of acidic vesicular organelles (AVOs), leading to the upregulation of beclin-1 and Atg7, a reduction in p62 expression (transcriptional), and the inactivation of PI3K/Akt signaling.	*In vitro*: cell cycle arrest In vivo: ↓tumor growth	86
	PN50G	Polysaccharide	*Pleurotus nebrodensis*	*In vitro*: A549 cell line	–	Induction of autophagy through the stimulation of AMPK activation. Nonetheless, it also impeded PI3K/Akt phosphorylation, suppressed mTOR activation, and enhanced the expression of beclin 1 and LC-3 in A549 cells.	*In vitro*: ↓cell proliferation *Invivo*: ↓tumor volume and weight	87
	P. Ferulae anti-tumor protein (PFAP)	Protein	*Pleurotus ferulae lanzi*	*In vitro*: A549 cell line *Invivo*: xenograft mice model	–	PFAP induces autophagy in A549 lung cancer cells by significantly activating AMPK, upregulating p62, LC3 II/I, and other related proteins, while inhibiting the mTOR pathway. This mechanism also reduced tumor growth in a xenograft mouse model.	*In vivo*: ↓tumor growth	88
	GMI	Protein	*Ganoderma microsporum*	*In vitro*: A549 cell line *In vivo*: mouse xenograft model	*In vitro*: GMI induced autophagy in 18% and 50% of A549 cells at 0.6 μmol/L and 1.2 μmol/L, respectively. *In vivo:* GMI administration significantly inhibited tumor growth and promoted autophagy in tumors *(P* < 0.05).	Autophagic cell death is induced, enhancing LC3 conversion and reducing p53 expression (transcriptional) in A549 lung cancer cells via a calcium-mediated signaling mechanism. In an A549 xenograft tumor model, this process reduced tumor formation and regulated autophagy *in vivo*.	*In vitro*: ↓cell proliferation *In vivo*: inhibition of tumor formation	89
			*Ganoderma microsporum*	*In vitro*: A549 cell line *In vivo*: mouse xenograft model	*In vitro*: After GMI treatment, the percentages of autophagic AVOs were 42.9% and 26% in the A549/D16 and A549/V16 sublines, respectively. *In vivo*: When compared with the group of mice administered PBS, the growth of tumors in the GMI group was significantly inhibited (*P* < 0.05).	GMI induces autophagy in A549 cells by inhibiting Akt/mTOR, offering a strategy to counteract multidrug resistance (MDR) in lung cancer. It also inhibits tumor growth *in vivo*, including those overexpressing p-glycoprotein (P-gp).	*In vitro*: ↑apoptotic death of MDR cell line *In vivo*: ↓growth of the MDR subline	90,91
			*Ganoderma microsporum*	*In vitro*: A549 and CaLu-1 cell line	Combined treatment with high-dose GMI (0.3 mmol/L) and bafilomycin-A1 caused severe cell damage and partially reduced the number of cancer cells containing autophagosomes.	Activation of autophagy in A549 and CaLu-1 cells by inhibiting PKB/mTOR, together with co-treatment with the lysosomal inhibitor bafilomycin-A1, resulted in substantial autophagosome production in CaLu-1/GFP-LC3 cells.	*In vitro*: autophagic cell death	92
	Latcripin-1	Triterpenoid.	*Lentinula edodes*	*In vitro*: A549 cancer cell line	–	The induction of autophagy enhances the appearance of autophagosomes in A549 cells, limits their maturation, modulates tumor cell growth, and initiates programmed cell death.	*In vitro*: ↓cell proliferation	93
Colorectal cancer	*G. lucidum* ribonuclease	Protein	*G. lucidum*	*In vitro*: HCT 116 *In vivo*: HT-29 xenografted mouse model	GLR suppressed autophagy in HT-29 and HCT 116 cells, as indicated by p62 accumulation, LC3-I upregulation, and LC3-II downregulation. pEGFP-LC3 labeling showed increased LC3 protein in GLR-treated HCT 116 and some HT-29 cells compared to controls.	Inhibited autophagy through the accumulation of P62, resulting in the overexpression of LC3-I and the downregulation of LC3-II.	*In vitro*: ↓cell proliferation *In vivo*: ↓tumor growth	98
	Lentinan	Polysaccharide	*Lentinus edodes*	*In vivo*: HT-29 cells and tumor-bearing non-obese diabetic (NOD)/severe combined immunodeficiency (SCID) mice		Directly reduced the growth of human colon cancer through ERS-mediated autophagy.	*In vivo*: ↓tumor volume and weight	103
Gastric cancer	Recombinant Lz-8	Protein	*G. lucidum*	*In vitro*: in SGC-7901 human gastric cancer cells	Autophagy played a key role in rLz-8-induced cell death *(p* < 0.05).	Induction of autophagy via endoplasmic reticulum stress is achieved by considerably elevating LC3 at both protein and mRNA levels (transcriptional).	*In vitro*: autophagic cell death	109
	Hispidin	Flavonoid	*Phellinus linteus*	*In vitro*: in SGC-7901 and GES-1 gastric cancer cells	Hispidin (122 μmol/L) induced necrotic cell death associated with autophagy in SGC-7901 cells.	Autophagy is induced through lysosomal membrane permeabilization by inhibiting tubulin polymerization.	*In vitro*: inhibition of autophagic and necrotic cell death	111
	N6-(2-hydroxyethyl)-adenosine	Nucleoside	*Cordyceps species*	*In vitro*: in SGC-7901 human gastric cancer cells *In vivo*: gastric cancer nude mouse model	*In vitro*: HEA (100 μmol/L) treatment upregulated ATG5, ATG12, and Beclin-1, while downregulating p62, indicating that HEA-induced autophagy contributed to apoptosis in SGC-7901 cells. *In vivo*: After 19 days of intragastric administration in BALB/c nude mice, HEA at 75 mg/kg and 100 mg/kg showed tumor inhibition rates of 54.66% and 64.90%, respectively.	Stimulates autophagy by enhancing the expression of LC3-II. The downregulation of p62 expression (transcriptional), alongside the elevation of *ATG5, ATG12*, and Beclin-1 levels, indicates that HEA-induced autophagy mediates apoptosis in SGC-7901 cells.	*In vitro*: cytotoxic effect *In vivo*: inhibition of tumor growth	112
	Latcripin 1	Triterpenoid	*Lentinula edodes*	*In vitro*: in SGC-7901 and BGC-823 human gastric cancer cell lines	Latcripin 1 treatment significantly increased the number of autophagic vacuoles in SGC-7901 and BGC-823 cells in a dose-dependent manner compared to controls (*P* < 0.05).	Autophagy is induced by the formation of autophagosomes and the conversion of light chain 3 (LC3-I to LC3-II).	*In vitro*: induction of cell autophagic and apoptotic cell death; inhibition of metastasis	113
	Latcripin-7A	Triterpenoid	*Lentinula edodes*	*In vitro*: in SGC-7901 and BGC-823 human gastric cancer cells	–	Autophagy is induced by inhibiting the PI3K/Akt/mTOR signaling pathway	*In vitro*: cell cycle arrest	127
Breast cancer	Polysaccharides (PS-T)		*Trametes robiniophila*	*In vitro*: human breast cancer MDA-MB-231 cells and mouse breast cancer 4T1 cells *In vivo*: six-week-old female BALB/c mice model	Autophagy was induced by inhibiting epithelial–mesenchymal transition *in vivo* in BALB/c female mice and *in vitro* in human MDA-MB-231 and murine 4T-1 breast cancer cells (*P* < 0.05).	PS-T treatment for 24 h increased the percentage of autolysosomes GFP⁻/RFP^+^ dots) compared to the control (*P* < 0.001).	*In vitro*: inhibition of cancer cell invasion and metastasis *In vivo*: anti-metastasis effect	122
	Lentinan	Polysaccharide	*Lentinus edodes*	*In vitro*: MCF-7 cells *In vivo:* BALB/c-nude mice	–	Induction of autophagy in neoplastic tissues through the analysis of autophagy marker proteins. LC3, p62, and Beclin-1 were examined in BALB/c-nu mice and MCF-7 cells.	*In vitro*: ↓cell proliferation *In vivo*: inhibition of tumor growth	126
	Latcripin-7A	Triterpenoid	*Lentinus edodes*	*In vitro*: MCF-7 and MDA-MB-231	LP-7A dose-dependently promoted autophagy in MCF-7 and MDA-MB-231 cells (*P* < 0.01).	Induction of autophagy through the upregulation of Beclin-1, Atg proteins, and LC3 I/II, alongside the downregulation of p62 expression (transcriptional) in MCF-7 and MDA-MB-231 cells.	*In vitro*: induction of cell autophagic and apoptotic cell death and inhibition of metastasis	114
	β-Glucan (LNT)		*Lentinus edodes*	*In vitro*: T47D cells	Autophagic cell death in human breast cancer T47D cells is induced by suppressing Nur77 expression, Akt/mTOR signaling, and inflammatory pathways.	LNT-20 mg/kg promoted autophagic cell death in breast cancer cells by inhibiting the expression of Akt/mTOR and nuclear factor-kappaB (NF-κB) signaling pathway-related proteins	*In vitro*: autophagic cell death	128
	Polysaccharide		*Ganoderma applanatum*	*In vitro*: MCF-7 cells	–	Induction of early autophagy in MCF-7 cells through the MAPK/ERK pathway	*In vitro*: inhibition of cancer cell growth	129
Liver cancer	Polysaccharide combined with vitamin C (GFP/VC)		*Grifola frondosa*	*In vitro*: SMMC-7721 and HepG2 cells	–	Autophagy is induced by overexpressing Beclin-1 and LC3 through the inhibition of PI3K and activation of JNK pathways.	*In vitro*: ↓cell proliferation	139
			*Grifola frondosa*	*Invivo:* BALB/c strain mice	Compared to the negative control, the GFP/VC group showed significantly higher expression of autophagy-related proteins Beclin-1 and LC3 (*P* < 0.05).	Beclin-1 and LC3 levels increased, promoting autophagy by inhibiting the PI3K pathway and activating JNK signaling.	*In vivo:* anti-tumor activity	138
	Agrocybe aegerita lectin	Protein	*Agrocybe aegerita*	*In vitro*: (HCC) cells	–	Stimulates autophagy by promoting the accumulation of LC3II, the formation of enhanced green fluorescent protein-tagged protein light chain (3EGFP-LC3) puncta, the development of AVOs, and the activation of autophagosomes.	*In vitro*: ↓cell viability	140
	Armillaridin	Triterpenoid	*Armillaria mellea*	*In vitro*: HepG2 HCC, HA22T, and Huh7 cell	–	Induction of autophagy through the aggregation of LC3 and the conversion of LC3-I to LC3-II	*In vitro*: ↓cell proliferation	141
	Eburicoic acid	Triterpenoid	*Antrodia cinnamomea*	*In vitro*: Hep 3B cells	Autophagosome and autophagic lysosomal content significantly increased with rising Eburicoic acid concentration (*P* < 0.05).	Autophagy is induced through the conversion of LC3-I to LC3-II, resulting in the creation of many autophagosomes/autolysosomes, alongside the downregulation of death-associated protein kinase (DAPK) and the upregulation of Beclin-1, JNK, and Bcl-2.	*In vitro*: ↓cell viability	142
Ovarian cancer	Grifolin	Triterpenoid	*Albatrellus Confluens*	*In vitro*: SKOV-3 and A2780 ovarian cancer cell lines	Flow cytometry with acridine orange staining confirmed that grifolin induced autophagy by increasing AVO formation dose-dependently (*P* < 0.05). Compared to controls, grifolin significantly upregulated LC3B, Atg7, and Beclin-1, while decreasing p62 in a dose-dependent manner.	Autophagy is stimulated by reduced proteins associated with the p-Akt, p-mTOR, p-p70S6K, and p-4E-BP1 pathways, while enhancing the autophagy markers LC3B, Atg7, and Beclin-1.	*In vitro*: induction of autophagic cell death	147
	Poricoic acid A (PAA)	Triterpenoid	*Poria cocos*	*In vitro*: SKOV-3 ovarian cancer cell line *In vivo*: nude mice xenograft model	*In vitro*: PAA induced autophagy and suppressed the mTOR/p70S6K signaling pathway. *In vivo*: PAA treatment reduced the tumor weight by nearly 50% (*P* < 0.01).	Autophagy is stimulated by restricting the mTOR/p70S6K signaling pathways and increasing the concentrations of the proteins LC3-I and LC3-II.	*In vitro*: suppression of cellular viability, migration, and invasion. *In vivo*: ↓tumor weight	148
	Adenosine derivative (CME)	Nucleoside analog	*Cordyceps militaris*	*In vitro*: A2780, OVCAR3, SKOV3, TOV112D, HEK293 human ovarian cancer cell lines *In vivo*: BALB/c nude mice	After 48 h, CME- and cordycepin-treated groups exhibited autophagic vacuoles, while the control group showed significantly fewer.	*In vitro*: Autophagy induced cellular destruction through the ENT1-AMPK-mTOR signaling cascade. *In vivo:* Autophagy is initiated due to cell membrane targeting by equilibrative nucleoside transporter 1 (ENT1), which activates AMPK and facilitates downstream protein targets.	*In vitro*: induction of autophagic cell death *In vivo*: ↓tumor volume	149
	Coenzyme Q	Benzoquinones	*Antrodia camphorate*	*In vitro*: SKOV-3 ovarian cancer cell line	Coenzyme Q dose-dependently increased the LC3-II accumulation in SKOV-3 cells.	Autophagy, triggered as a survival mechanism, was evidenced by increased accumulation of LC3-II, the formation of GFP-LC3 puncta, the creation of AVOs, and dysregulation of beclin-1/Bcl-2, which were linked to the inhibition of HER-2/neu and PI3K/Akt signaling pathways.	*In vitro*: autophagic cancer cell survival	150
Glioblastoma	Cordycepin	Nucleoside analog	*Cordyceps* ssp.	*In vitro*: SH-SY5Y and U-251 cell lines	–	Induction of autophagy by increasing LC3I/II mRNA (transcriptional) and protein	*In vitro*: cell survival	156
	Cordycepin and cordycepic acid	Nucleoside analog	*Cordyceps militaris*	*In vitro*: GBM8401 and U-87MG cells	Mycelial fermentation (MF) reduced mTOR expression but increased Atg5 and LC3 II levels in GBM8401 cells.	Induction of autophagy by downregulating Bcl-2	*In vitro*: induction of cell death and disruption of the cell cycle	157
	Coenzyme Q0	Benzoquinones	*Antrodia camphorate*	*In vitro*: U87MG and GBM8401 cells	–	Induction of autophagy through the inhibition of PI3K/Akt/mTOR signaling pathways drastically altered tumor xenografts by promoting autophagy and reducing tumor burden in xenografted athymic nude mice.	*In vitro*: induction of cell death	158
Skin cancer	GA-DM	Triterpenoid	*G. lucidum*	*In vitro*: HT-144, 1359-mel, and DM-331 cells *In vivo*: murine B16 melanoma model	GA-DM induced autophagy associated with cell survival at early time points (3–6 h) in treated cells (*P* < 0.01).	Autophagy was induced in human melanoma cell lines HT-144, 1359-mel, and DM-331 by enhancing the expression of Beclin-1 and LC-3. Additionally, GA-DM triggered a potential link between autophagy and apoptosis.	*In vitro*: autophagic cell death *Invivo:* tumor weight	160
Prostate cancer	Cordycepin	Nucleoside analog	*Cordyceps militaris*	*In vitro*: LNCaP human prostate cancer cell line		Autophagy is stimulated by the increased accumulation of LC3-II and widespread formation of LC3 puncta in the LNCaP human prostate cancer cell line.	*In vitro*: cell survival	162
	Resveratrol	Stilbene	*Pleurotus florida*	*In vitro*: DU145 (HTB-81) and PC3 (CRL1435) prostate cancer cell lines.	Resveratrol (100 μmol/L) treatment in prostate cancer cells significantly induced ER stress and autophagy by inhibiting the mTOR/Akt pathway.	Autophagy is triggered by a decrease in endoplasmic reticulum (ER) calcium storage and store-operated calcium entry (SOCE), leading to ER stress, which activates AMPK and inhibits the Akt/mTOR pathway.	*In vitro*: induction of autophagic cell death	163
	GA-DM	Triterpenoid	*G. lucidum*	*In vitro*: LNCaP human prostate cancer cell line	GA-DM-treated PC-3 cells expressed higher levels of Beclin-1, Atg-5, and LC3-II proteins.	Autophagy is inhibited by the disruption of autophagosome and lysosome fusion, preventing the formation of autophagic vacuoles, particularly LC3-II, during the pre-apoptotic phase.	*In vitro*: cell death induction (initial stage) and survival mechanism (under severe ER stress).	58
Leukemia	Poricoic acid A	Water-soluble polysaccharide	*Xiankongjun* Taiwan, China	*In vitro*: THP-1 cells	Rapamycin partially reduced WSPIS-induced annexin-V-positive cells at 48 h from 15.70% in controls to 9.74% with WSPIS treatment and also decreased WSPIS-induced cell death (*P* < 0.01).	Inhibition of autophagy in THP-1 cells blocks the conversion of cytosolic LC3-I to membrane-associated LC3-II, leading to decreased levels of LC3-II.	*In vitro*: cell cycle arrest and EndoG-mediated apoptosis	167
		Triterpenoid	*Poria cocos*	*In vitro*: T-cell acute lymphoblastic leukemia (T-ALL) cells	–	Autophagy is activated in T-cell acute lymphoblastic leukemia (T-ALL) cells through the modulation of the AMPK/mTOR and LC3 signaling pathways.	*In vitro*: ↓cell viability and cell cycle arrest.	166
Pancreatic cancer	4-Acetylantroquinonol B	Quinonoid	*Niuzhangzhi*	*In vitro*: MiaPaCa 2 cell line	4-AAQB treatment significantly downregulated autophagy-associated proteins in MiaPaCa-2 and MiaPaCa-2GEMR cells (*P* < 0.05).	Autophagy is suppressed by downregulating the PI3K/Akt/MDR1 pathway in gemcitabine-resistant pancreatic cancer cells, enhancing therapeutic sensitivity.	*In vitro*: ↑chemosensitivity	168
	Fudan-Yueyang	Polysaccharides	*G. lucidum*	*In vitro*: PANC-1 and BxPC-3 cells	FYGL dose-dependently promoted autophagosome formation in the PANC-1 cell line.	Autophagy is inhibited by blocking the fusion of autophagosomes and lysosomes.	*In vitro*: cell death	169
	Antroquinonol	Quinonoid	*Antrodia camphorata*	*In vitro*: PANC-1 and AsPC-1 human pancreatic carcinoma cells	–	Suppression of autophagy by reducing mTOR activity via the inhibition of the PI3-kinase/Akt pathway.	*In vitro*: cell cycle arrest and mitochondrial apoptosis	170
Head and neck cancer	YMGKI-1 (3-[4-(3-Methylbut-2-enyloxy)phenyl]-4-isobutyl-N-hydroxypyrrole-2,5-dione)	Substituted hydroxypyrrole-2,5-dione	*Antrodia cinnamomea*	*In vitro*: Head and neck cancer-initiating cells (HN-CICs)	Treatment with YMGKI-1 at 10, 25, and 35 μg/mL induced autophagy in approximately 12%, 20%, and 43% of HN-CICs, respectively.	Autophagic cell death is triggered in head and neck cancer-initiating cells (HN-CICs) through the activation of AMPK and downregulation of the PI3K–mTOR pathway. Furthermore, AVO formation and the LC3-II/LC3-I ratio increase in a dose-dependent manner.	*In vitro*: autophagic cell death	172
	Coenzyme Q0 (2,3-dimethoxy-5-methyl-1,4-benzoquinone)	Benzoquinone	*Antrodia camphorata*	*In vitro*: FaDu-TWIST1 cell *line* *Invivo*: nude mice Xenografted nude mice	–	Autophagic cell death is induced in FaDu-TWIST1 cells through the accumulation of LC3-II and the formation of acidic vesicular organelles (AVOs). This effectively delays and reduces tumor occurrence and burden in FaDu-TWIST1-xenografted nude mice.	*In vitro*: ROS-mediated cell death, metastasis inhibition *Invivo:* tumor suppression	173
Bladder cancer	FIP-gts	Protein	*Ganoderma tsugae*	*In vitro*: N/P (cisplatin-resistant sub-line) urothelial cancer cells	–	Induction of autophagy-mediated, caspase-independent cell killing through significant autophagosome accumulation and LC3-II activation in N/P (cisplatin-resistant sub-line) urothelial carcinoma cells.	*In vitro*: caspase-independent cell death	174
Esophageal cancer	Ganoderic acid D	Triterpenoid	*G. lucidum*	*In vitro*: EC9706 and Eca109 esophageal squamous cell carcinoma (ESCC) cells	–	Autophagic cell death is enhanced in EC9706 and Eca109 esophageal squamous cell carcinoma (ESCC) cells by downregulating phosphorylated PI3K, Akt, and mTOR proteins in the mTOR signaling pathway, coupled with increased autophagosome formation	*In vitro*: cell proliferation and autophagic cell death	175

Note: Mechanisms described as “increased/decreased expression” or “up/downregulation” refer to protein levels unless stated otherwise. Transcriptional changes are explicitly noted. AMPK: AMP-activated protein kinase; Atg7: Autophagy-related protein 7; AVO: Acidic vesicular organelle; A549: Human lung adenocarcinoma cell line; BAX: Bcl-2-associated X protein; Bcl-XL: B-cell lymphoma-extra large; Beclin-1: Autophagy-related protein; DAPK: Death-associated protein kinase; EGFP: Enhanced green fluorescent protein; EndoG: Endonuclease G; ENT1: Equilibrative nucleoside transporter 1; ERK: Extracellular signal-regulated kinase; ERS: Endoplasmic reticulum stress; GMI: *Ganoderma microsporum* immunomodulatory protein; HER-2/neu: Human epidermal growth factor receptor 2; HN-CICs: Head and neck cancer-initiating cells; JNK: c-Jun N-terminal kinase; LC3: Microtubule-associated protein 1A/1B-light chain 3; MAPK: Mitogen-activated protein kinase; MDR: Multidrug resistance; mTOR: Mechanistic target of rapamycin; NCI-H460: Human large cell lung carcinoma cell line; NF-κB: Nuclear factor kappa-light-chain-enhancer of activated B cells; PFAP: *P. ferulae* anti-tumor protein; P-gp: P-glycoprotein; PKB/Akt: Protein kinase B; p62/SQSTM1: Sequestosome 1; p53: Tumor protein p53; p70S6K: Ribosomal protein S6 kinase beta-1; ROS: Reactive oxygen species; SPCA-1: Human lung adenocarcinoma cell line; SOCE: Store-operated calcium entry; WSPIS: Water-soluble polysaccharide from *Xiankongjun* Taiwan, China; 4E-BP1: Eukaryotic translation initiation factor 4E-binding protein 1; -: Not available.

**Table 3:** Comparative study of the mushrooms in different cancers with their translational potentials.

Mushroom name	Extract/bioactive compound	Cancer types	Potency indicators	Specificity	Translational potential	Reference
*G. lucidum*	Triterpenes, fruiting body, polysaccharides	Lung, colorectal gastric, breast, liver, prostate, pancreatic, skin, esophageal	- Induces autophagy via p38 inhibition and Beclin-1/LC3 upregulation - Reduces tumor growth *in vivo*	High (targets p38, mTOR, and MAPK/ERK pathways across cancers)	High	27,49,82,97,98,107,109,124,136,160,169,175,
*Cordyceps militaris*	Cordycepin, adenosine derivatives, nano-extracts	Lung, prostate, glioblastoma, ovarian skin (wound healing)	- Inhibits mTOR and promotes LC3/Atg5 expression - ENT1–AMPK–mTOR signaling (ovarian) - c-FLIPL degradation - *In vivo* tumor suppression - Autophagic cell death induction	High (ENT1–mTOR cascade, FLIPL degradation, mTOR inhibition, and AMPK activation) Works in drug-resistant models	High	85,102,149,157,161,162
*Antrodia salmonea*	Ethanolic/aqueous extracts	Colon, breast, ovarian, head and neck	- Triggers ROS-mediated autophagy/apoptosis - Suppresses tumor volume *in vivo*	Moderate (ROS-dependent; affects Akt/mTOR, NF-κB, β-catenin)	High	100,123,146,171,176
*Antrodia cinnamomea*	Ethanolic extracts (ACF2)	Colon, breast	- Activates CHOP/TRB3/Akt/mTOR pathway - Induces autophagic cell death	High (specific to *CHOP/TRB3/ULK1* axis)	Moderate	99,142,172,177
*Trametes robiniophila(Huaier)*	Polysaccharides	Breast	- Inhibits mTOR/S6K pathway - Reduces tumor growth *in vivo*	Moderate (mTOR-focused; effective in triple-negative breast cancer)	Moderate	122
*Inonotus obliquus(Chaga)*	Ethanol extract	Breast	- Activates AMPK–mTOR pathway - Suppresses tumor growth *in vivo*	Moderate (AMPK/mTOR axis; broad breast cancer applicability)	Moderate	120
*Grifola frondosa(Maitake)*	Cold-water extract	Liver	- Inhibits PI3K/JNK pathways - Reduces tumor growth *in vivo*	High (*PI3K*/JNK-specific; targets hepatocellular carcinoma)	Moderate	137, 138, 139

AMPK: AMP-activated protein kinase; Akt: Protein kinase B; Atg5: Autophagy-related protein 5; c-FLIPL: Cellular FLICE-like inhibitory protein; CHOP: C/EBP homologous protein; ENT1: Equilibrative nucleoside transporter 1; JNK: c-Jun N-terminal kinase; LC3: Microtubule-associated protein 1A/1B-light chain 3; MAPK/ERK: Mitogen-activated protein kinase/Extracellular signal-regulated kinase; mTOR: Mechanistic target of rapamycin; NF-κB: Nuclear factor kappa-light-chain-enhancer of activated B cells; PI3K: Phosphoinositide 3-kinase; p38: p38 mitogen-activated protein kinase; ROS: Reactive oxygen species; S6K: Ribosomal protein S6 kinase; TRB3: Tribbles homolog 3; ULK1: Unc-51-like autophagy activating kinase 1.

### Lung cancer

**Figure 3:**
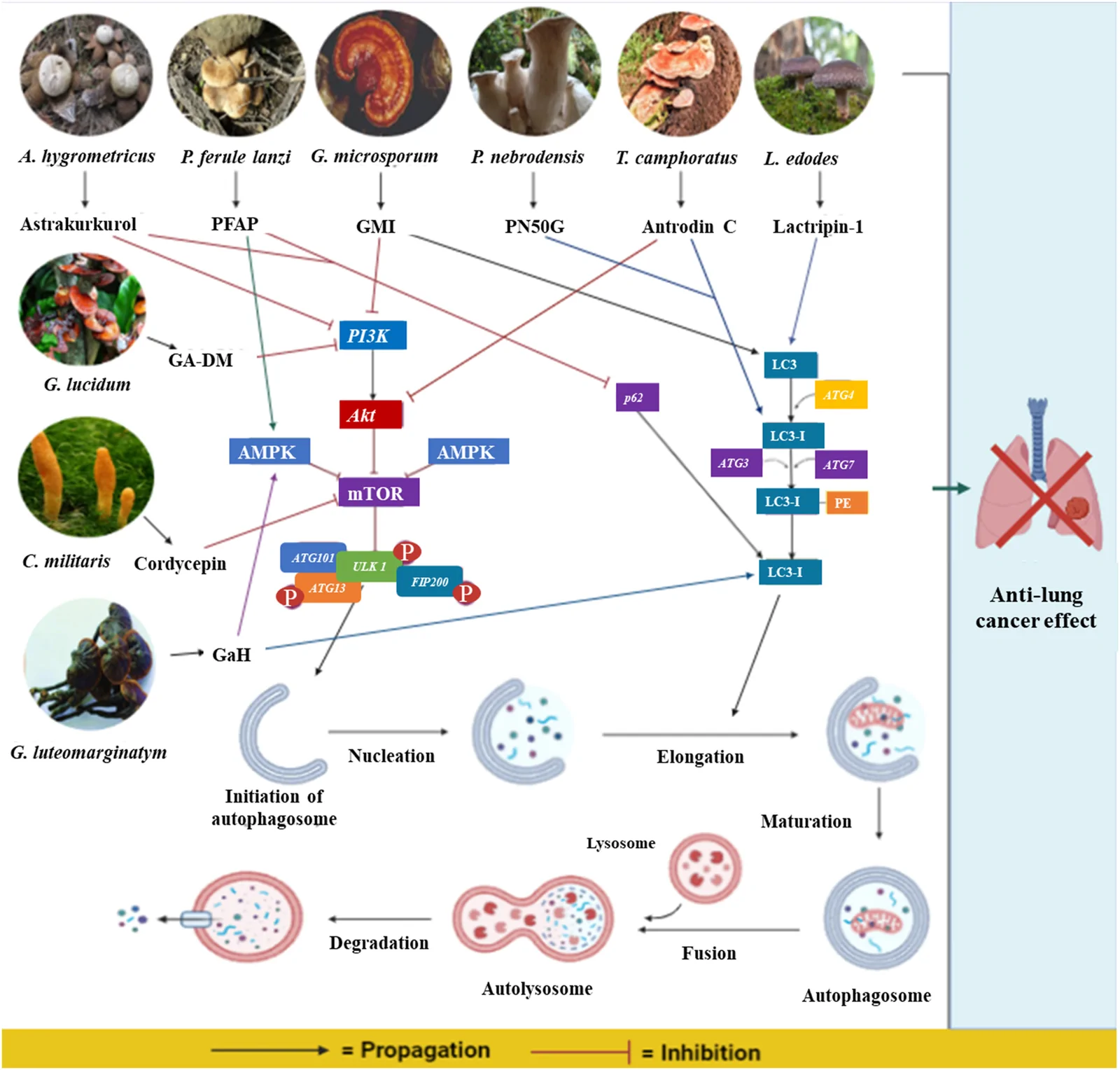
Mechanistic illustration of mushrooms and their bioactive compounds in lung cancer via autophagy modulation. Medicinal mushrooms such as *L. edodes*, *G. lucidum*, and *C. militaris* induce autophagy in NSCLC cells by targeting the PI3K/Akt/mTOR and AMPK pathways. Their bioactive constituents modulate key autophagy markers, enhancing autophagic flux and demonstrating potential as anticancer agents. Akt: Protein kinase B; AMPK: AMP-activated protein kinase; LC3-II: Microtubule-associated protein 1A/1B-light chain 3, form II; mTOR: Mechanistic target of rapamycin; PI3K: Phosphoinositide 3-kinase; ULK1: Unc-51-like autophagy activating kinase 1.

Lung cancer is the primary cause of cancer-related mortality worldwide, accounting for 18.4% of all such deaths. In the US, an estimated 236,740 new cases and 130,180 deaths from lung cancer were projected for 2022.^81^ Recent studies have demonstrated that various autophagy-modulating compounds isolated from mushrooms can inhibit lung cancer cell proliferation through multiple signaling pathways [Figure 3].^16^

GA-DM, a compound from *G. lucidum*, inhibits the proliferation of A549 and NCI–H460 NSCLC cells by activating autophagy and apoptosis via the PI3K/Akt/mTOR pathway. Autophagy plays a pro-apoptotic role, as its suppression diminishes the apoptotic effects of GA-DM.^82^ GaH, a natural prostanoid isolated from *G. luteomarginatum,* blocked the proliferation of A549 and H1299 cells in a time- and concentration-dependent manner. It activated AMPK and upregulated the expression of p53, Beclin-1, and LC3B, thereby inducing autophagy in A549 and H1299 lung cancer cell lines.^83^ Antrodin C, from *T. camphoratus*, reduced proliferation and invasion in SPCA-1 cells by activating autophagy through the Akt-mTOR pathway, independent of AMPK. Autophagy inhibition with chloroquine increased apoptosis, suggesting autophagy had a protective role.^84^ Chen et al. also reported the cytoprotective role of autophagy in A549 lung cancer cells treated with antrodin C.^85^ Cordycepin (the primary active component of *C. militaris*) significantly reduced the cell viability of H1792, H1299, H460, H157, and A549 cell lines in a dose-dependent manner, where it activated pro-apoptotic autophagy and caused autophagy-induced disruption of cellular FLICE-like inhibitory protein, long isoform (c-FLIPL).^86^ Astragurkurol, a terpenoid from *Astraeus hygrometricus*, inhibits A549 lung cancer cell growth and migration by inducing autophagy (via AVO formation, beclin-1 and Atg7 upregulation, p62 reduction, and PI3K/Akt pathway inactivation). Autophagy inhibition reduced apoptosis, showing its protective role. It also suppressed tumor progression in an *ex vivo* xenograft model.^87^ PN50G, a polysaccharide from *P. nebrodensis*, inhibited A549 cell proliferation and reduced tumor volume and weight in a dose-dependent manner. It induced autophagy by upregulating beclin 1 and promoting LC3-I to LC3-II conversion.^88^ PFAP, an anti-tumor protein from *Pleurotus ferulae lanzi*, suppresses mTOR in NSCLC A549 cells, activating autophagy and increasing the expression of P62, LC3 II/I, and related proteins. It also demonstrated tumor growth reduction in a xenograft mouse model *in vivo.* GMI, a protein from *G. microsporum*, triggered autophagic cell death in A549 lung cancer cells by enhancing LC3 conversion, reducing p53, and activating a calcium-mediated pathway. It also inhibited tumor growth and regulated autophagy in an A549 xenograft model *in vivo*.^89^ GMI inhibited the PI3K/Akt/mTOR pathway, induced autophagy and apoptosis in multidrug-resistant lung cancer cells, and reduced tumor growth in mice xenografts via autophagy and apoptosis, independent of p-glycoprotein overexpression.^90^^,^^91^ Yang et al. showed that GMI primarily activated autophagy through the PKB pathway in A549 and CaLu-1 lung cancer cells, with mTOR playing a role in GMI-induced autophagy.^92^ Latcripin-1 (LP1), a novel antitumor mycoprotein from *Lentinula edodes,* halted the growth and induced autophagy of A549 cells.^93^

### Colorectal cancer

**Figure 4:**
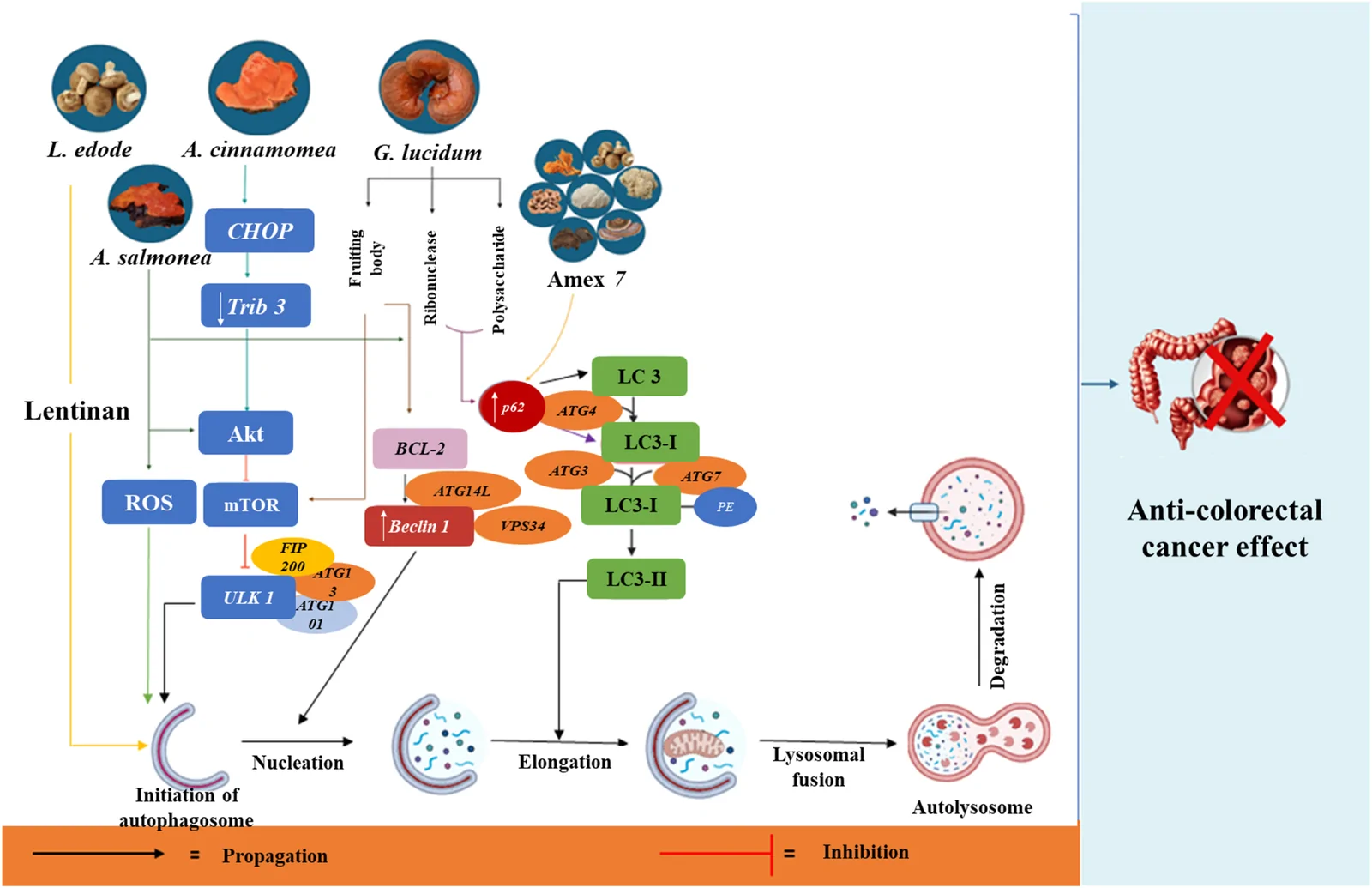
Modulation of autophagy in colorectal cancer by mushroom-derived bioactives. *Antrodia cinnamomea* extract induces autophagy in HCT 116 cells by inhibiting the CHOP/TRB3/Akt/mTOR pathway, increasing CHOP and TRB3 levels, and decreasing LC3-II. *A. salmonea* elevates p62, ROS, the Beclin-1/Bcl-2 ratio, and LC3-II levels, while reducing Akt/mTOR activity. GLR, GLP, GLE, and Amex7 from *G. lucidum* enhance autophagy by increasing p62 and LC3-II. Lentinan (SLNT) from *L. edodes* promotes ER stress and autophagy. CHOP: C/EBP homologous protein; GLE: *G. lucidum* extract; GLP: *G. lucidum* polysaccharide; GLR: *G. lucidum* ribonuclease; LC3-I/II: Microtubule-associated protein 1A/1B-light chain 3, form I/II; ROS: Reactive oxygen species; TRB3: Tribbles pseudokinase 3; ULK1, Unc-51-like autophagy activating kinase 1.

Colorectal cancer (CRC) is the third most common cancer worldwide and the second leading cause of cancer-related deaths, with over 1.8 million new cases and 881,000 deaths reported in 2018. Its incidence is projected to rise by 60% by 2030.^94^ Several autophagy-modulating compounds isolated from various mushrooms have been shown to inhibit the growth of colorectal cancer cells through diverse signaling pathways [Figure 4].^95^

*G. lucidum* triterpene extract (GLT) suppressed the progression of human colon cancer cells (HT-29) and inhibited xenograft tumor growth via autophagy induction. This was accomplished by inhibiting the p38 mitogen-activated protein kinase (MAPK) pathway, increasing Beclin-1 and LC3 expression, and reducing p38 MAPK phosphorylation, thereby achieving cancer inhibition through autophagy.^96^ The fruiting body extract of *G. lucidum* (GLE) induced autophagy in HCT 116 colorectal cancer cells by modulating key autophagy markers (Beclin-1, LC3B/LC3A, p-mTOR, *ATG5*, and total mTOR). This reduced cell proliferation *in vitro* and decreased tumor weight and volume *in vivo*, contributing to cancer suppression.^97^ *G. lucidum* ribonuclease (GLR) suppressed autophagy in HCT 116 and HT-29 cells by increasing p62, upregulating LC3-I, and downregulating LC3-II, inducing apoptosis and inhibiting cancer cell proliferation. This demonstrates that *G. lucidum* compounds can differentially modulate autophagy.^98^ Moreover, *G. lucidum* polysaccharide (GLP) induced autophagy in cancer cells by increasing LC3-II levels and autophagosome formation while disrupted autophagic flux by blocking autophagosome-lysosome fusion. This blockage led to autophagosome accumulation, triggering apoptosis via the MAPK/ERK pathway and inhibiting tumor growth.^95^ Besides, *Antrodia cinnamomea* showed potent anticancer effects in the CRC cell line HCT 116. It induced autophagic cell death by upregulating the endoplasmic reticulum stress marker CHOP and its downstream gene *TRB3*, leading to dephosphorylation of Akt and mTOR and a significant suppression of the tumor growth *in vivo* in CRC.^99^ *Antrodia salmonea* induced autophagy in SW620 colon cancer cells by inhibiting Akt/mTOR signaling, *NF-κB, β-catenin* expression, and modulating autophagy markers (LC3-II accumulation, *p62/SQSTM1* activation, *ATG4B* inactivation, AVO formation, and Beclin-1/Bcl-2 disruption), enhancing autophagy with a cytoprotective effect.^100^ Selenium nanoparticles derived from *Pleurotus tuber-regium* induced autophagy in HCT 116 CRC cells by elevating LC3-II and beclin-1 levels and reducing *p62*/*SQSTM1* expression, leading to cancer cell death and highlighting their potential in anti-CRC therapy.^101^ Amex7, a combination of extracts from seven medicinal mushrooms (*Phellinus linteus, Grifola frondosa, Hericium erinaceum, Lentinula edodes, Sparassis crispa, Trametes versicolor*, and *Cordyceps militaris)*, elevated p62 and LC3A/B-II expression in HT-29 cells, promoting autophagy and enhancing its anticancer effect.^102^ Lentinan, derived from *Lentinus edodes*, induced autophagy in HT-29 cells by reducing p62 and increasing LC3-II levels, mediated by endoplasmic reticulum stress. This led to autophagic cell death, inhibiting CRC in cell and animal models.^103^

### Gastric cancer

**Figure 5:**
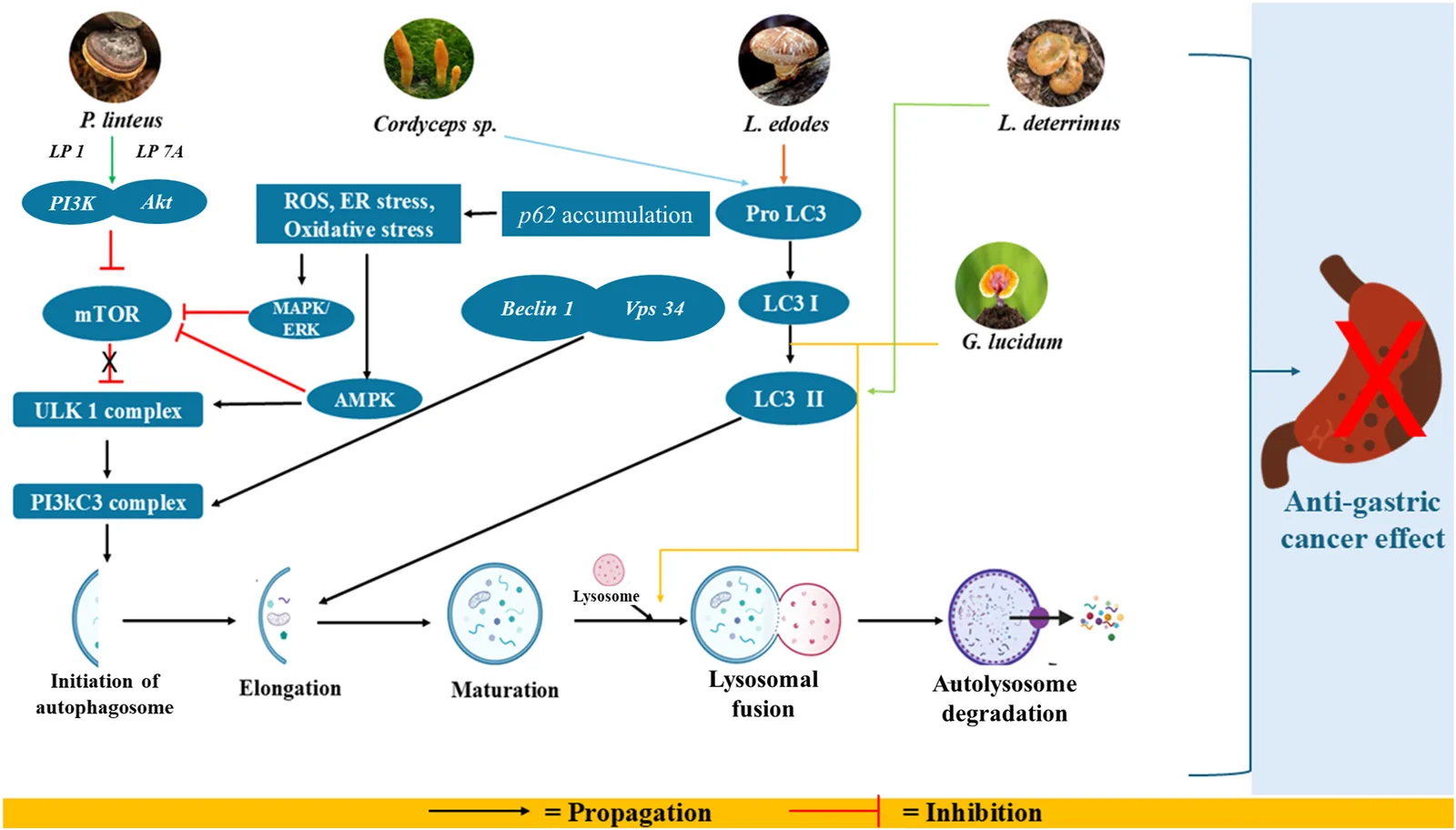
Mechanistic illustration of mushrooms and their bioactive compounds in gastric cancer via autophagy modulation. *P. linteus* inhibits mTOR through the PI3K/Akt pathway, initiating autophagosome formation. *Cordyceps* sp., *L. edodes*, and *L. deterrimus* enhance LC3-II accumulation, promoting autophagosome elongation. *G. lucidum* modulates autophagy by acting on LC3-II levels and facilitating lysosomal fusion with autophagosomes. Akt: Protein kinase B; AMPK: AMP-activated protein kinase; LC3-II: Microtubule-associated protein 1A/1B-light chain 3, form II; mTOR: Mechanistic target of rapamycin; PI3K: Phosphoinositide 3-kinase; ULK1: Unc-51-like autophagy activating kinase 1.

Gastric cancer (GC), a leading epithelial malignancy originating in the stomach, is characterized by its biological complexity and influenced by numerous risk factors.^104^ In 2020, it accounted for approximately 1.09 million new cases and 0.77 million deaths globally.^105^ Currently, GC ranks as the third leading cause of cancer-related deaths and the fifth most prevalent cancer worldwide.^106^ Several studies have shown that mushrooms can inhibit GC by inducing autophagy, serving as a potential mechanism of cancer suppression [Figure 5].

A methanolic extract of *G. lucidum* fruiting bodies inhibited the growth of human GC AGS cell lines by upregulating the autophagy marker LC3-II and enhancing autophagosome formation, as evidenced by monodansyl cadaverine tagging.^27^ Moreover, the same methanolic extract further induced autophagy in AGS gastric tumor cells by promoting autophagosome formation, enhancing LC3-II levels, and reducing p62 expression, indicating the autophagy-mediated reduction of cell growth.^107^ Similarly, a cold methanolic extract (−20 °C) of *G. lucidum* induced autophagy in AGS cells by increasing autophagosome formation, contributing to cancer inhibition.^108^ Recombinant Lz-8, derived from *G. lucidum*, induced autophagic cell death through endoplasmic reticulum stress in human GC cells (SGC-7901). This was evidenced by an increase in LC3 levels, at the protein and mRNA levels, supporting its role in cancer suppression through autophagy.^109^ Additionally, the ethanolic extract of *Lactarius deterrimus* (LDE) inhibited the growth of AGS cells while triggering cytoskeleton rearrangements that led to autophagy, further suppressing cancer progression.^110^ Hispidin, a phenolic compound isolated from Phellinus linteus, induces autophagic and necrotic cell death in SGC-7901 and GES-1 cells via lysosomal membrane permeabilization by inhibiting tubulin polymerization, further promoting cancer cell death.^111^ Furthermore, N6-(2-hydroxyethyl)-adenosine, a derivative from *Cordyceps* species, promoted autophagic cell death in SGC-7901 cells by upregulating LC3-II, downregulating p62, and increasing the levels of *ATG5, ATG12*, and Beclin-1, further indicating autophagy-mediated apoptosis and cancer suppression.^112^ LP1, derived from *Lentinula edodes*, also triggered autophagy in human GC cells by promoting the formation of autophagosomes and converting LC3I into LC3II, contributing to cancer suppression.^113^ Additionally, latcripin-7A, another peptide from *Lentinula edodes*, inhibited the growth of SGC-7901 and BGC-823 cells by suppressing the PI3K/Akt/mTOR pathway, thus inducing autophagy and cancer inhibition.^114^

### Breast cancer

**Figure 6:**
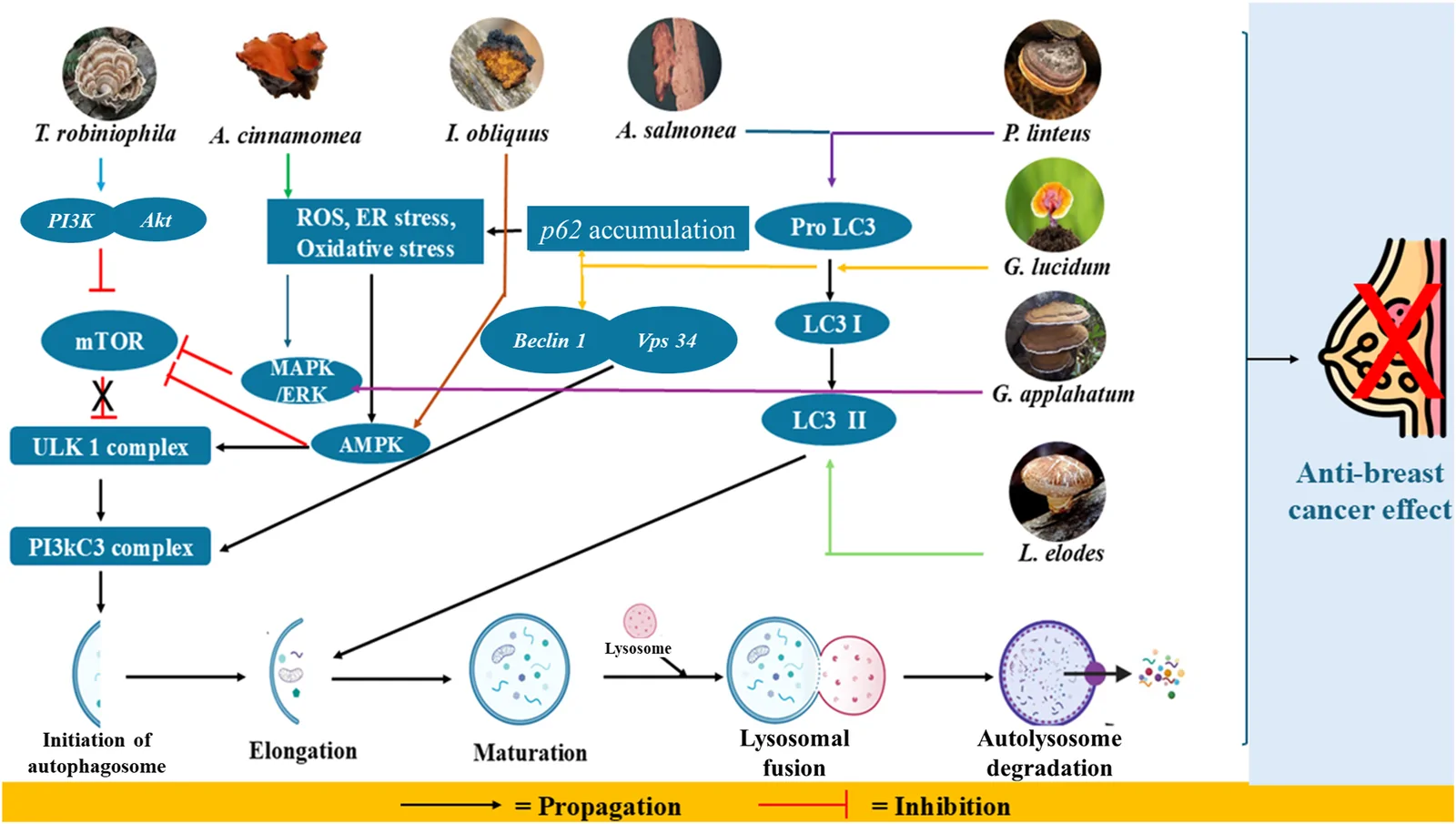
Mechanistic illustration of mushrooms and their bioactive compounds in breast cancer via autophagy modulation. *mTOR* inhibition by *I. obliquus* and *T. robiniophila* triggers autophagy, leading to cell death. ER stress induced by *A. cinnamomea* initiates autophagy, while *A. salmonea* and *P. linteus* enhance autophagy through LC3 upregulation and induce G2 phase arrest. *G. lucidum* and *P. linteus* promote autophagy by modulating p62, LC3, and Beclin-1. *L. edodes* and its constituents regulate LC3, while *G. applanatum* targets the MAPK/ERK pathway, collectively promoting autophagy and suppressing breast cancer progression. AMPK: AMP-activated protein kinase; ER: Endoplasmic reticulum; LC3: Microtubule-associated protein 1A/1B-light chain 3; MAPK/ERK: Mitogen-activated protein kinase/extracellular signal-regulated kinase; mTOR: Mechanistic target of rapamycin; ULK1: Unc-51-like autophagy activating kinase 1.

Breast cancer is one of the most prevalent cancers globally, accounting for approximately 25% of all cancer cases in women and contributing to about 15% of cancer-related deaths among women.^115^^,^^116^ In 2023, the United States reported an estimated 300,590 new cases and 43,700 deaths due to breast cancer.^117^ Autophagy plays a dual role in breast cancer: it supports tumor survival or induces cell death depending on the cellular context.^118^ Notably, mushrooms with autophagic-modulating properties effectively inhibit breast cancer cell growth [Figure 6].

The ethanolic extract of Chaga mushroom (*Inonotus obliquus*), widely used in food and medicine, induces autophagy in breast cancer cell lines MDA-MB-231, MDA-MB-468, and MCF-7 by inhibiting the mTOR/S6K pathway.^119^ The extract stimulated AMPK activation, causing mTOR inhibition, a vital controller of cell growth and proliferation. This suppression induced autophagy, promoting the breakdown of cellular materials and potentially reducing cancer cell growth and survival.^120^ *Trametes robiniophila (Huaier)* suppresses the mTOR/S6K signaling pathway, triggering autophagy and degrading cellular components. This process reduces tumor growth and promotes autophagic cell death, emphasizing Huaier’s potential as a supportive treatment.^121^ Furthermore, polysaccharides derived from *Trametes robiniophila Murr* exhibited tumor-suppressing effects by inducing autophagy. This process led to the degradation of *Snail*, a critical transcription factor involved in epithelial-mesenchymal transition (EMT). Polysaccharides promoted autophagy, suppressed EMT, and inhibited cancer cell invasion and metastasis, as demonstrated *in vivo* in BALB/c mice and *in vitro* in MDA-MB-231 and 4T-1 cancer cells.^122^ *Antrodia salmonea* inhibited cancer progression by inducing autophagy and apoptosis. In MDA-MB-231 cells, it increased LC3-II, upregulated *ATG7*, inhibited mTOR, causing mitochondrial dysfunction, and suppressed tumor growth in xenografted nude mice.^123^ Moreover, *A. salmonea* demonstrated its potential to prevent cancer by inducing autophagy, as evidenced *in vitro* and *in vivo*. The findings indicated that *A. salmonea*-induced cytotoxicity involved G2 cell-cycle arrest in MDA-MB-231 cells, characterized by a reduction in cyclin B1, A, and E, as well as CDC2 proteins. The prevention of this cell-cycle arrest by N-acetylcysteine (NAC) suggested that ROS accumulation played a significant role.^99^ Additionally, *A. salmonea* treatment decreased *COX-2* expression and induced Poly (ADP-ribose) polymerase (PARP) cleavage, which NAC pretreatment reversed, further supporting the involvement of oxidative stress in *A. salmonea*-mediated cell cycle regulation and tumor suppression in xenografted mice. *G. lucidum*, dissolved in DMSO, induced autophagy in MCF-7 and MDA-MB-231 cells by increasing Beclin-1, LC3, and p62 levels, inhibiting tumor cell proliferation and causing G2 cell cycle arrest, thereby suppressing tumor growth.^124^ Similarly, combining a small dosage of 5-fluorouracil (5-FU) with ethanol extract from the fruiting bodies of *Phellinus linteus*, another medicinal mushroom, enhanced the autophagic response in human triple-negative breast cancer (MDA-MB-231) cells. This combination induced cell death through autophagy, enhanced the conversion of LC3-I to LC3-II, promoted the formation of acidic vesicular organelles (AVOs), and facilitated the visualization of numerous double-membraned vacuoles at the ultrastructural level. Combining *Phellinus linteus* with 5-FU presents a promising strategy for enhancing autophagy-mediated tumor suppression by inhibiting cell proliferation and inducing cell cycle arrest in therapy.^125^ Additionally, *Lentinus edodes* showed significant treatment effects through its bioactive polysaccharide Lentinan, which induced autophagy by modulating LC3, p62, and Beclin-1 in tumor tissues of BALB/c-nu mice and MCF-7 cells.^126^ Additionally, latcripin-7A, another bioactive compound from *Lentinus edodes*, promoted autophagy by increasing the expression of Beclin-1, ATG proteins, and LC3 I/II while decreasing p62 levels in MCF-7 and MDA-MB-231 cells.^127^ Notably, Yu et al. demonstrated that the bioactive component β-Glucan, derived from the same mushroom, induced autophagic cell death in human breast cancer T47D cells by suppressing *Nur77* expression, inhibiting Akt/mTOR signaling, and modulating inflammatory pathways.^128^ Collectively, *Lentinus edodes* suppresses breast cancer via autophagy, apoptosis, and inflammation modulation. Similarly, *Ganoderma applanatum’s* polysaccharides activate MAPK/ERK signaling, inducing autophagy in MCF-7 cells, suppressing tumor growth, and demonstrating its therapeutic potential.^129^

### Liver cancer

Liver cancer ranks as the sixth most common cancer worldwide and has a broad geographic distribution, particularly in sub-Saharan Africa, Eastern and Southeast Asia, and Melanesia.^130^^,^^131^ According to GLOBOCAN 2020, primary liver cancer cases are projected to increase by 55%, with a 56.4% rise in related deaths by 2040.^132^ Mushrooms have been shown to influence liver cancer through the modulation of autophagy, a process that can either suppress tumor development or support cancer cell survival under stress.^133^

An extract from *Hypsizygus marmoreus* fruiting bodies induced autophagy-mediated death in Hep 3B liver cancer cells by converting LC3-I to LC3-II and upregulating p62, demonstrating autophagy’s tumor-suppressive role through cellular degradation.^134^ Moreover, *Inonotus baumii* extract activates autophagy in liver cancer cells (SMMC-7721) and nude mice via the AMPK/mTOR/ULK1 pathways, increasing LC3-II and decreasing p62, leading to autophagic cell death and tumor growth inhibition.^135^

Furthermore, *G. lucidum* spore powder extract exhibited significant anticancer effects in HepG2 and Huh6 cells, as well as in BALB/c mice, by suppressing RACK1 (receptor for activated C kinase 1) O-GlcNAcylation. This suppression led to reduced RACK1 expression and modulated autophagy, malignancy, and immune responses, highlighting the extract’s potential as a therapeutic agent for hepatoblastoma.^136^ Moreover, cold water extraction from fresh fruiting bodies of *Grifola frondosa* induced autophagy in 5-week-old male BALB/c athymic nude mice, as well as in human hepatocellular carcinoma (HCC) cell lines Hep3B, HA22T, and Huh7. This effect was mediated through the activation of JNK pathways and the inhibition of PI3K, causing suppressed cell proliferation *in vitro* and reduced tumor growth *in vivo.*^137^^,^^138^ Moreover, *Grifola frondosa* polysaccharide (GEP) combined with vitamin C induced autophagy in SMMC-7721 and HepG2 hepatoma cells by increasing Beclin-1 and LC3II expression and inhibiting the PI3K/Akt/mTOR/p70S6K signaling pathways.^139135^ Additionally, GFP isolated from the same mushroom induced autophagy in BALB/c mice when combined with vitamin C. Here, autophagy was implicated in cancer suppression by disrupting survival pathways and promoting the degradation of cancer cells.^138^ Moreover, *Agrocybe aegerita* lectin induced autophagy in liver cancer by promoting LC3-II accumulation, EGFP-LC3 puncta formation, AVO development, and autophagosome creation. This autophagy enhanced apoptosis, suppressing cancer cell viability and contributing to tumor growth reduction.^140^ Furthermore, armillaridin, present in the edible and medicinal mushroom *Armillaria mellea*, caused autophagy by promoting LC3 aggregation and the conversion of LC3-I to LC3-II in HepG2 HCC, HA22T, and Huh7 cells, which caused cell death.^141^ Eburicoic acid from *Antrodia cinnamomea* fruiting bodies suppressed liver cancer by inducing autophagy. It activated phosphorylation of Beclin-1, JNK, and Bcl-2 pathways, triggering ER stress-mediated cell death and promoting autophagic degradation of damaged cancer cells, reducing proliferation.^142^

### Ovarian cancer

Ovarian cancer (OC) ranks as the seventh most common cancer among women worldwide. It is often asymptomatic in its early stages, making it difficult to detect, and is associated with a high mortality rate, ranking among the deadliest gynecologic cancers alongside uterine and cervical cancers. OC primarily affects women aged 65 and older.^143^ Globally, it is estimated to cause over 150,000 deaths annually, while in the US, approximately 13,270 deaths were projected in 2023.^144^^,^^145^

Yang et al.^146^ demonstrated that bioactive compounds—ergostanes, lanostanes, naphthoquinones, and polyphenols—from the hyphae of *Antrodia salmonea* aqueous extracts triggered autophagy-mediated cell death in OC cells (SKOV-3 and A2780). This was evidenced by increased LC3-II, GFP-LC3 puncta, and AVO formation, along with p62 activation, *ATG4B* suppression, *ATG7* upregulation, and Beclin-1/Bcl-2 disruption, thereby inhibiting cancer progression and autophagy resistance.^146^ Similarly, grifolin, a secondary metabolite isolated from the edible mushroom *Albatrellus confluens* (Northern truffle), stimulates autophagy-mediated tumor suppression in OC cell lines SKOV-3 and A2780 by inhibiting key components of the Akt/mTOR pathway, including p-Akt, p-mTOR, p-p70*S6K*, and p-4E-BP1, while upregulating autophagic biomarkers LC3B, ATG7, and Beclin-1.^147^ Ma, Rui et al. found that poricoic acid A from *Poria cocos* inhibits mTOR/p70*S6K* signaling, increases LC3-I/II levels, promotes autophagy, and suppresses viability, migration, and invasion of SKOV-3 cells, besides reducing tumor weight in mice.^148^ However, in human OC cell lines A2780, OVCAR3, SKOV3, TOV112D, and HEK293, cordycepin, an adenosine derivative extracted from *C. militaris*, can cause autophagic cellular destruction through the equilibrative nucleoside transporter 1 (ENT1)-AMPK-mTOR signaling cascade. Additionally, the methanolic extract of *C. militaris*, along with cordycepin, demonstrates *in vivo* efficacy in BALB/c nude mice models by activating AMPK via ENT1 transport.^149^ This activation leads to downstream autophagic cell death, thereby contributing to the suppression of ovarian cancer. Notably, Coenzyme Q from *Antrodia camphorata* induces autophagy in SKOV-3 cells as a cytoprotective response, marked by increased LC3-II, GFP-LC3 puncta, AVOs, and Beclin-1/Bcl-2 disruption. This pro-survival autophagy may reduce CoQ’s therapeutic efficacy by helping cancer cells resist apoptosis.^150^

### Cervical cancer

Cervical cancer is the fourth most common cancer in women and the third leading cause of cancer-related deaths in low- and middle-income countries.^151^ The United States recorded an expected 13,820 new cases and 4360 deaths of cervical cancer by 2024.^152^

For instance, the aqueous extract of *Sanghuangporus baumii* inhibits tumor growth in U14 cervical cancer cells in female Kunming mice by upregulating autophagy-related genes (*GABARAP*, *VMP1*, *VAMP8*, and *STX17*) and increasing LC3II/LC3I ratios.^153^ The ethanolic extract of *Lenzites betulina* promotes autophagy in HeLa cells, causing cell cycle arrest, reduced invasion, and tumor growth inhibition; its compound 4′’-hydroxy-6-methoxyaurone reduces drug resistance by targeting P-glycoprotein.^154^ Additionally, water-soluble extracts from the mycelium of *Pleurotus ostreatus* and *Pleurotus eryngii* induce autophagic cell death in SiHa cervical cancer cells via the ER stress–mitochondrial pathway.^155^ These mushrooms utilize various autophagy mechanisms to suppress cervical cancer progression.

### Glioblastoma

In glioblastoma, mushroom-derived compounds like cordycepin, cordycepic acid, and Coenzyme Q10 regulate autophagy differently. Cordycepin from *Cordyceps* activates autophagy in SH-SY5Y and U-251 cells, promoting cancer cell survival by helping them evade apoptosis through increased LC3I/II expression.^156^ Conversely, *Cordyceps* militaris and its mycelial fermentation induce autophagy and apoptosis in GBM8401 and U-87MG glioblastoma cells by downregulating Bcl-2 and inhibiting the Akt-mTOR pathway, using autophagy to suppress cancer and promote cell death.^157^ Similarly, Coenzyme Q0 from *Antrodia camphorata* induces autophagy and apoptosis in U87MG and GBM8401 glioblastoma cells by inhibiting the PI3K/Akt/mTOR pathway and triggering ROS-mediated cytotoxicity.^158^ Thus, although cordycepin initially promotes cancer cell survival by regulating autophagy, *Cordyceps militaris* and Coenzyme Q10 exploit autophagy to induce apoptosis and suppress glioblastoma, showcasing the dual role of autophagy in cancer progression and therapy.

### Skin cancer

Mushroom-derived compounds suppress skin cancer by regulating autophagy. *Trametes versicolor* extract induces autophagy in SK-MEL-5 melanoma cells, increasing LC3-II and immune checkpoint expression while inhibiting migration.^159^ GA-DM from *G. lucidum* promotes autophagy and apoptosis in melanoma cells by boosting Beclin-1 and LC3.^160^ *Cordyceps militaris*, delivered via nanoparticles, stimulates autophagy to reduce oxidative stress and support fibroblast regeneration, potentially inhibiting cancer growth.^161^

### Prostate cancer

Cordycepin induces autophagy in LNCaP prostate cancer cells, as evidenced by increased LC3 puncta formation, accumulation of LC3-II, and enhanced autophagic flux. When autophagic flux is blocked by bafilomycin A1, the cells undergo apoptotic cell death, suggesting that cordycepin-induced autophagy functions as a survival mechanism in these cancer cells.^162^ Resveratrol, a bioactive compound derived from the fruiting bodies of *Pleurotus florida*, induces autophagy-mediated cell death in prostate cancer cell lines DU145 and PC3. This effect occurs through the depletion of ER calcium stores and inhibition of store-operated calcium entry (SOCE), leading to AMPK activation and suppression of the AKT/mTOR signaling pathway.^163^ GA-DM, a compound from *G. lucidum*, suppresses prostate cancer by simultaneously inducing autophagy and apoptosis via *Beclin-1*, *Atg5* upregulation, along with modulation of the *Bax*/*Bcl-2* ratio. It also enhances immune responses through the upregulation of HLA class II molecules. However, under conditions of ER stress, excessive autophagy may paradoxically support tumor survival.^164^

### Leukemia

*Ganoderma tsugae* ethanolic extracts induce protective autophagy in K562 leukemia cells by increasing LC3-II expression, disrupting the Beclin-1/Bcl-2 complex, and promoting the formation of AVOs. While this suppresses cell viability, it may also contribute to cancer cell survival through autophagy.^165^ In contrast, poricoic acid A from *Poria cocos* induces autophagy in T-cell acute lymphoblastic leukemia (T-ALL) cells by modulating the AMPK/mTOR and *LC3* signaling pathways, causing reduced cancer cell viability and cell cycle arrest.^166^ Additionally, a polysaccharide extract from *Xiankongjun* Taiwan, China inhibits autophagy in THP-1 leukemia cells by decreasing LC3-II levels. This inhibition enhances WSPIS-induced apoptosis and prevents leukemia cells from utilizing autophagy as a survival mechanism.^167^

### Pancreatic cancer

Inhibiting autophagy using mushroom-derived compounds shows promise for treating advanced pancreatic cancer, where cells rely on autophagy for survival and chemotherapy resistance. 4-Acetylantroquinonol B (4-AAQB) from *Niuzhangzhi*, Fudan-Yueyang from *G. lucidum*, and antroquinonol from *Antrodia camphorata* inhibit autophagy and enhance cancer cell death. 4-AAQB also boosts gemcitabine efficacy by blocking receptor for advanced glycation end-products (RAGE)/HMGB1-mediated PI3K/Akt/MDR1 signaling in *MiaPaCa-2* cells.^168^ Although Fudan-Yueyang prevented autophagosome-lysosome fusion in *PANC-1* and *BxPC-3* cells, leading to apoptosis.^169^ Antroquinonol blocked mTOR activity through the PI3K/Akt pathway in *PANC-1* and *AsPC-1* cells, inducing apoptosis and senescence.^170^ Although autophagy initially suppresses tumors by maintaining homeostasis, it later contributes to cancer growth and therapy resistance. Targeting autophagy inhibition with mushroom-derived compounds suppresses pancreatic cancer progression and improves conventional treatment efficacy, offering a promising therapy.

### Head and neck cancer

In head and neck cancer, *Antrodia salmonea* suppresses tumor growth by regulating autophagy. In head and neck squamous cell carcinoma (HNSCC) cells with Twist overexpression, *A. salmonea* fermented broth increases LC3-I/II, acidic AVO formation, and p62 expression, promoting autophagy-induced apoptosis and reducing tumor size in xenografted mice.^171^ For example, the medicinal mushroom *Antrodia cinnamomea* contains YMGKI-1, a maleic and succinic acid derivative isolated from its mycelia. YMGKI-1 induces autophagic cell death in head and neck cancer-initiating cells (HN-CICs) by activating AMPK and inhibiting the PI3K-mTOR pathway. This causes a dose-dependent increase in AVOs and an elevated LC3-II/LC3-I ratio, thereby promoting autophagic cell death.^172^ Additionally, *Antrodia camphorata* produces coenzyme Q10, promoting autophagic cell death in FaDu-TWIST1 cells via LC3-II accumulation and AVO formation, reducing tumor growth in mice.^173^

### Urothelial cancer

In urothelial cancer, *Ganoderma tsugae*’s FIP-gts combined with chloroquine induces autophagy-dependent, *caspase*-independent cell death, causing autophagosome accumulation and LC3-II activation, resensitizing cisplatin-resistant cancer cells to treatment.^174^

### Esophageal cancer

Finally, in esophageal squamous cell carcinoma (ESCC), ganoderic acid D from *G. lucidum* induces autophagic cell death in EC9706 and Eca109 cells by downregulating phosphorylated proteins in the PI3K/Akt/mTOR pathway and enhancing autophagosome formation, offering a promising therapeutic approach through synergistic autophagy and apoptosis.^175^

## Safety evaluation of key compounds

Mushroom-derived compounds modulate autophagy in cancer but face clinical challenges such as safety and variable efficacy. Cordycepin shows dose-dependent hepatotoxicity, inducing autophagic death in lung cancer but promoting survival in prostate cancer.^145^^,^^146^^,^^148^^,^^157^^,^^158^^,^^160^ GA-DM demonstrates selective cytotoxicity but may promote tumor survival under severe endoplasmic reticulum stress, particularly in prostate models.^160^^,^^147^ Antrodin C induces cytotoxic autophagy in colorectal cancer while exhibiting cytoprotective effects in lung adenocarcinoma, which diminishes chemotherapy sensitivity.^114^^,^^132^ Coenzyme Q from *Antrodia camphorata* shows low toxicity but promotes ovarian cancer survival via autophagy.^150^^,^^143^ Conversely, hispidin shows limited efficacy in TP53-mutant gastric cancers despite inducing lysosomal permeabilization. Lentinan unexpectedly enhances transforming growth factor-β signaling in triple-negative breast cancer, although poricoic acid A promotes tumor growth in immunosuppressed pancreatic cancer models. The identified contradictions, frequently arising from genetic heterogeneity, autophagy flux thresholds, and variations in extract purity, underscore the necessity for standardized dosing, genetic stratification, and combinatorial strategies to enhance therapeutic applications.

## Limitations and future perspective

This review underscores the potential of medicinal mushrooms to modulate autophagy as a strategy for cancer prevention, while emphasizing limitations such as insufficient research and dependence on crude extracts, which compromise the clarity and reproducibility of observed anticancer mechanisms. The use of whole mushrooms or unrefined extracts, rather than purified bioactive compounds and standardized formulations, hinders consistency, reproducibility, and the establishment of reliable dose-response relationships.

A major gap exists in comprehensive clinical trial data and large-scale epidemiological studies needed to substantiate the efficacy, optimal dosing, and safety of mushroom-based interventions for cancer prevention and treatment in humans. Additionally, translating promising *in vitro* and *in vivo* findings into effective clinical applications remains challenging due to issues such as poor bioavailability, interspecies metabolic differences, and the complexity of human cancer biology compared to model systems. Addressing these limitations is essential for accurately assessing the therapeutic potential of medicinal mushrooms in oncology.

While the modulation of autophagy by mushroom-derived compounds presents a promising avenue for cancer therapy, progress requires coordinated multidisciplinary efforts. Key priorities include standardizing extract preparation, identifying and characterizing active constituents, elucidating mechanisms of action, and conducting rigorous clinical trials to evaluate efficacy and safety. Establishing robust cultivation, extraction, and quality control protocols is critical to ensure the consistency, potency, and safety of mushroom-derived products used in clinical settings.

Advanced researches employing phytochemical and molecular biology techniques are needed to clarify how these compounds influence autophagy and interact with key cancer signaling pathways. Exploring the synergistic potential of combining mushroom-derived autophagy modulators with conventional chemotherapeutics, targeted therapies, synthetic analogs, and immunotherapies may offer a comprehensive and integrative approach to cancer treatment. Such combination strategies could involve administering standardized mushroom extracts as adjunct oral agents alongside conventional treatments, with dosing regimens aligned to therapeutic cycles to enhance efficacy and reduce adverse effects.

Despite encouraging preclinical evidence, the clinical translation of mushroom-derived therapeutics remains in early stages. Well-designed clinical trials are imperative to validate therapeutic efficacy, determine optimal dosing strategies, and assess safety across various cancer types and stages. Personalized treatment approaches, tailored to individual genetic and metabolic profiles, may further enhance therapeutic outcomes and minimize adverse effects.

## Conclusion

Medicinal mushrooms have long been recognized for their health benefits and are now undergoing rigorous scientific evaluation for their anticancer properties. Their growing prominence in cancer therapy is largely attributed to a diverse array of bioactive compounds. This review synthesizes evidence from multiple studies demonstrating that various polysaccharides and chemical constituents—such as GA-DM and cordycepin—derived from different mushroom species, induce autophagic cell death, thereby reducing cancer cell viability and proliferation.

Extracts from mushrooms, including *G. lucidum*, *Antrodia salmonea*, and *Antrodia cinnamomea,* have shown promising effects in tumor suppression, enhanced survival rates, and improved immune responses. Notably, mushroom-derived compounds have exhibited selective cytotoxicity, promoting autophagic cell death in cancer cells while sparing normal cells, highlighting their potential as targeted therapeutic agents.

The development of mushroom-based cancer therapeutics faces several key challenges. These include the standardization of extract preparation, isolation, and characterization of active compounds, and integration of these compounds with conventional chemotherapeutic regimens. Furthermore, well-designed clinical trials are essential to validate the safety and efficacy of these agents in oncology.

If these challenges are effectively addressed, mushroom-derived autophagy modulators may become a valuable adjuvant component of clinical cancer treatment, offering a natural and effective means to complement existing modalities and improve patient outcomes.

## Authors contribution

Md. Mahmudul Hasan: conceptualization, writing–original draft, data extraction, and data analysis, review and editing, ; Eva Azme: conceptualization, writing–original draft, data extraction, and data analysis, review and editing; Rashedul Alam: conceptualization, writing–original draft, data extraction, and data analysis, review and editing; Md. Jahirul Islam Mamun: writing–original draft; Md. Tanvir Chowdhury: writing–original draft; Md. Hossain Rasel: writing–original draft; Md. Safayat Hossen Momen: writing–original draft; Neamul Hoque: writing–original draft; Md. Ekramul Haque Ekram: writing–original draft; Nazmul Hasan Eshaque: writing–original draft; Shakil Ahmed: writing-original draft; Md. Tashrif Rahman Tipu: writing–original draft; Sanjida Shahid Juthi: writing-original draft; Mohammad Fazlul Kabir: writing–original draft; Ahsan Ullah: writing–original draft; Md. Liakot Ali: conceptualization, writing-original draft, manuscript revision. S.M. Moazzem Hossen and Hea-Jong Chung: Conceptualization, supervision, project administration, and manuscript revision. All the authors critically revised and approved the final version of the manuscript.

## Ethics statement

None.

## Data availability statement

The datasets used in the current study are available from the corresponding author on reasonable request.

## Declaration of Generative AI and AI-assisted technologies in the writing process

The authors declare that generative artificial intelligence (AI) and AI assisted technologies were not used in the writing process or any other process during the preparation of this manuscript.

## Funding

None.

## Conflict of interest

The authors declare that they have no known competing financial interests or personal relationships that could have appeared to influence the work reported in this paper.
